# Tumor microenvironment evaluation promotes precise checkpoint immunotherapy of advanced gastric cancer

**DOI:** 10.1136/jitc-2021-002467

**Published:** 2021-08-10

**Authors:** Dongqiang Zeng, Jiani Wu, Huiyan Luo, Yong Li, Jian Xiao, Jianjun Peng, Zilan Ye, Rui Zhou, Yunfang Yu, Gaofeng Wang, Na Huang, Jianhua Wu, Xiaoxiang Rong, Li Sun, Huiying Sun, Wenjun Qiu, Yichen Xue, Jianping Bin, Yulin Liao, Nailin Li, Min Shi, Kyoung-Mee Kim, Wangjun Liao

**Affiliations:** 1 Department of Oncology, Southern Medical University Nanfang Hospital, Guangzhou, Guangdong, China; 2 State Key Laboratory of Oncology in South China, Collaborative Innovation Center for Cancer, Sun Yat-sen University Cancer Center, Guangzhou, Guangdong, China; 3 Department of Oncology, The Second Affiliated Hospital of Guangzhou University of Chinese Medicine, Guangdong Provincial Hospital of Chinese Medicine, Guangzhou, Guangdong, China; 4 Department of Medical Oncology, The Sixth Affiliated Hospital, Sun Yat-sen University, Guangzhou, Guangdong, China; 5 Center of Gastrointestinal Surgery, The First Affiliated Hospital, Sun Yat-sen University, Guangzhou, Guangdong, China; 6 Department of Medical Oncology, Phase I Clinical Trial Centre, Sun Yat-sen Memorial Hospital, Sun Yat-sen University, Guangzhou, China; 7 Department of Dermatology, Johns Hopkins School of Medicine, Baltimore, Maryland, USA; 8 Department of Cardiology, State Key Laboratory of Organ Failure Research, Nanfang Hospital, Southern Medical University, Guangzhou, Guangdong, China; 9 Karolinska Institutet Department of Medicine-Solna, Clinical Pharmacology Group, Karolinska University Hospital, Stockholm, Sweden; 10 Department of Pathology and Translational Genomics, Samsung Medical Center, Gangnam-gu, Seoul, South Korea

**Keywords:** tumor microenvironment, computational biology, gastrointestinal neoplasms, immunotherapy

## Abstract

**Background:**

Durable efficacy of immune checkpoint blockade (ICB) occurred in a small number of patients with metastatic gastric cancer (mGC) and the determinant biomarker of response to ICB remains unclear.

**Methods:**

We developed an open-source TMEscore R package, to quantify the tumor microenvironment (TME) to aid in addressing this dilemma. Two advanced gastric cancer cohorts (RNAseq, N=45 and NanoString, N=48) and other advanced cancer (N=534) treated with ICB were leveraged to investigate the predictive value of TMEscore. Simultaneously, multi-omics data from The Cancer Genome Atlas of Stomach Adenocarcinoma (TCGA-STAD) and Asian Cancer Research Group (ACRG) were interrogated for underlying mechanisms.

**Results:**

The predictive capacity of TMEscore was corroborated in patient with mGC cohorts treated with pembrolizumab in a prospective phase 2 clinical trial (NCT02589496, N=45, area under the curve (AUC)=0.891). Notably, TMEscore, which has a larger AUC than programmed death-ligand 1 combined positive score, tumor mutation burden, microsatellite instability, and Epstein-Barr virus, was also validated in the multicenter advanced gastric cancer cohort using NanoString technology (N=48, AUC=0.877). Exploration of the intrinsic mechanisms of TMEscore with TCGA and ACRG multi-omics data identified TME pertinent mechanisms including mutations, metabolism pathways, and epigenetic features.

**Conclusions:**

Current study highlighted the promising predictive value of TMEscore for patients with mGC. Exploration of TME in multi-omics gastric cancer data may provide the impetus for precision immunotherapy.

## Background

Clinical trials of immune checkpoint blockade (ICB), antibodies, such as anti-programmed cell death protein 1 (PD-1) and anti-programmed death-ligand 1 (PD-L1), showed manageable toxicity and antitumor activity in patients with advanced gastric cancer (GC) in the ATTRACTION-2 and KEYNOTE-059 trials.^1 2^ However, different studies with ICB treatment revealed a highly variable objective response rate, ranging from 10% to 26% in patients with GC.^1 3 4^ Hence, the precise biomarkers to discriminate potential responders to immune therapies remains an urgent priority.

The biomarkers predictive of ICB response are under investigation. Currently, PD-L1 combined positive score (CPS), microsatellite instability-high (MSI-H), and tumor mutation burden (TMB) are widely recognized as promising biomarkers suggest greater efficacy of ICB despite some limitations.^5 6^ Immunohistochemistry (IHC)-based PD-L1 CPS, is most adopted but controversial for the PD-L1 expression heterogeneity, unstandardized detective process, and various positive criteria.^7^ Besides, ATTRACTION-2 suggested that the survival benefit with nivolumab in GC was independent of PD-L1 positivity (<1% vs ≥1%), indicating that PD-L1 positivity might omit part of responders.^1^ Patients with high TMB have a higher chance of mobilizing host immune reaction, thus responding to ICB, but facing several measurement hurdles.^8–10^ Likewise, MSI-H leads to the accumulation of somatic mutations and is rarely detected in patients with GC.^11 12^ The common ground of these biomarkers is the focus on the inherent characteristics of tumor cells and the neglection of the interactions with the tumor microenvironment (TME) components,^13^ thus partially interpreting unsatisfactory results in GC clinical trials exploring predictive biomarkers towards ICB.

The TME comprizing various immune cells, stromal cells, and extracellular components, profoundly affects tumorigenesis, progression, and therapeutic resistance.^14–17^ Increasing evidence indicated the implication of TME in the antitumor process, which can facilitate ICB response prediction.^15 18^ Researches reveal that a fraction of cancer-associated fibroblasts (CAFs), myeloid-derived suppressor cells, and macrophages can hijack ICB immunotherapy.^6 17 19^ Additionally, the TME stromal signals of the epithelial–mesenchymal transition (EMT)-related gene signature and transforming growth factor-beta (TGF-β)^6 20^ restrain antitumor immunity and response to ICB. However, ways to integrate these parameters lack full exploration, hindering optimizing selection strategies for potential ICB responders. Obstacles include an inaccurate combination of these parameters and uncertain interactions of these signatures.

Investigating the multi-omics data of 1524 patients with GC, we previously established a methodology termed TMEscore^15^ to evaluate the immune cell infiltration pattern. TMEscore is promising in determining the responsiveness to ICB in melanoma and metastatic urothelial cancer. For improvement, we optimized the TMEscore evaluation and verified its clinical utility in advanced gastric cancer using NanoString technology.^18 21 22^ We incorporated our TME-evaluation methodology into an open-source R package, TMEscore, to predict tumor immunogenicity and ICB sensitiveness from bulk transcriptomic data. To understand the TMEscore-related tumor intrinsic characteristics and antitumor immunity, we comprehensively analyzed the genomic characteristics, molecular subtypes, metabolic, and methylation features. The genomic and molecular biomarkers of response and resistance to ICB we identified demonstrates the complex host-tumor interplay in treatment response.

## Methods

### Human gastric cancer specimens and NanoString gene expression analysis

Formalin-fixed paraffin-embedded or fresh-frozen tumor tissue from multiple clinical centers was collected retrospectively at baseline before receiving checkpoint immunotherapy. Tumor responses were evaluated according to RECIST V.1.1 criteria. Tumor specimens derived from patients with mGC (up to 90 days from treatment start) were conducted as previously described by Ayers *et al*.^21^ Of 70 specimens from five clinical centers (Nanfang Hospital of Southern Medical University, Sun Yat-sen University Cancer Center, Guangdong Provincial Hospital of Chinese Medicine, The Sixth Affiliated Hospital of Sun Yat-sen University and The First Affiliated Hospital of Sun Yat-sen University), 48 specimens were of sufficiently high quality for RNA evaluation. A minimum of approximately 80 ng of total RNA was used to measure the expression of 51 TMEscore genes, comprizing 25 TME signature A genes, 19 TME signature B genes and some checkpoint-related genes (eg, *PD-L1*, *LAG3*, *PDCD1LG2*, *CTLA4*, *TIGIT*, *TIM3* and *PDCD1*), and 10 housekeeping genes (*ACTB*, *ABCF1*, *B2M*, *G6PD*, *GAPDH*, *GUSB*, *PGK1*, *RPLPO*, *TFRC* and *TUBB*) using the nCounter platform (NanoString Technologies; Seattle, Washington, USA).^22^ Data was normalized using the housekeeping genes.

### Gastric cancer specimens derived from clinical trial

Prospective, open-label, phase 2 trial (NCT02589496) of advanced gastric cancer was designed as a single-arm, phase 2 study at Samsung Medical Center. Immune checkpoint inhibitor (pembrolizumab) 200 mg was administered as 30 min intravenous infusion every 3 weeks until documented disease progression, unacceptable toxicity, or up to 24 months. Tumor responses were evaluated every two cycles according to RECIST V.1.1 criteria. Toxicities were graded based on the National Cancer Institute Common Terminology Criteria for Adverse Events V.4.0. Tumor sample collection, eligibility criteria, PD-L1 IHC, MSI status determination, Epstein-Barr virus (EBV) in situ hybridization, tissue genomic analysis, and RNA sequencing pipeline of this cohort were detailed in our previous research.^5^

### Other patient cohorts used in this study

Patient cohorts used in this study are summarized in online supplemental table S1. Seven genomic and transcriptomic data sets from patients with metastatic urothelial cancer treated with an anti-PD-L1 agent (NCT02951767),^6^ patients with metastatic melanoma and non-small-cell lung cancer treated with MAGE-3 agent-based immunotherapy (NCT00706238),^23^ patients with advanced melanoma treated with PD-1 blocker,^24^ patients with advanced melanoma treated with various types of immunotherapy from The Cancer Genome Atlas of Skin Cutaneous Melanoma (TCGA-SKCM) cohort,^25^ patients with melanoma treated with anti-CTLA-4 (cytotoxic T-lymphocyte-associated protein 4) or PD-1 (programmed cell death protein 1) antibody,^26^ and mouse model treated with anti-CTLA-4^27^ were downloaded and analyzed to determine the predictive capacity of TMEscore and were compared with its counterparts.

10.1136/jitc-2021-002467.supp1 Supplementary data

### TMEscore evaluation, immune cell deconvolution and signature score estimation

For the gene expression (normalized by RMA, TPM, FPKM or housekeeping genes) matrix, the expression of each gene in a signature was standardized so that its mean expression was 0, and the SD was 1 across samples. Then, PCA was performed, and principal component 1 was extracted to serve as the gene signature score. This approach had the advantage of focusing the score on the set with the largest block of well-correlated (or anti-correlated) genes in the set, while down-weighting contributions from genes that do not track with other set members.^6 15^ As our previous study^15^ indicated, TMEscore of each patient was estimated by the formula: TMEscore = ∑ PC1_i_ – ∑PC1_j_, where *i* is the signature score of clusters whose Cox coefficient is positive, and *j* is the expression level of the gene whose Cox coefficient is negative. The analytic code and package used to perform the TMEscore estimation are provided for non-commercial use at GitHub: https://githubcom/DongqiangZeng0808/TMEscore. To characterize the metabolism, immune microenvironment and other prevalent gene signatures activation in each tumor sample, multi-algorithms were applied to determine the pathway activity using IOBR package (https://github.com/IOBR/IOBR).^28^ ImmuneScore, Stromalscore, and tumor purity were assessed computationally in RNA-seq data using the ESTIMATE algorithm^29^ that uses gene expression signatures to infer the fraction of stromal and immune cells in tumor samples. Other computational algorithms and tools used to estimate the microenvironment were detailed in the online supplemental methods.

10.1136/jitc-2021-002467.supp3 Supplementary data

### Differentially gene expression analysis

All differential gene analyses were conducted using the DESeq2 package.^30^ Differential gene expression analysis was performed using a generalized linear model with the Wald statistical test, with the assumption that underlying gene expression count data were distributed per a negative binomial distribution with DESeq2. DEGs were considered for further analysis with a q value<0.05. The adjusted p value for multiple testing was calculated using the Benjamini-Hochberg correction.^31^

### Identification of TMEscore relevant mutations and mutational signatures

The mutation MAF files were downloaded with TCGAbiolinks,^32^ and the mutation status and mutation burden were inferred from the MAF files. Mann-Whitney U test was adopted to define the significance of binary variables (wild type or mutated). We applied the Benjamini-Hochberg method to convert the p values to adjusted p values.^31^ The mutational signature analysis was performed using the deconstructSigs package^33^ in R, which selects combinations of known mutational signatures^34^ that account for the observed mutational profile in each sample.

### Functional and pathway enrichment analysis

Gene annotation enrichment analysis was performed with the R package clusterProfiler.^35^ Enrichment p values were based on 1000 permutations and subsequently adjusted for multiple testing using the Benjamini-Hochberg procedure to control the false discovery rate (FDR).^31^ Gene Ontology (GO) and KEGG terms were identified with a strict cut-off of p<0.01 and an FDR of less than 0.05. We also identified pathways that were up-regulated and down-regulated among groups by running a gene set enrichment analysis (GSEA)^36^ of the adjusted expression data for all transcripts.

### Single-sample gene-set enrichment analysis of tumor processes

To characterize the tumor processes and pathway activation status in each tumor sample, a ssGSEA algorithm^37^ was applied to determine the pathway activity using GO,^38^ KEGG^39^ and HALLMARK gene sets derived from MSigDB (V.6.2).^40^ Other prevalent gene signature scores with respect to the TME, tumor intrinsic pathway, and metabolism were calculated for each sample using the PCA algorithm by IOBR package.^28^

### Differentially methylated probes analysis

Methylation data (β values of Illumina Infinium HumanMethylation450) of The Cancer Genome Atlas of Stomach Adenocarcinoma (TCGA-STAD) patients were obtained through TCGAbiolinks.^32^ β values reported by the 450K Illumina platform for each probe were set as the methylation level measurement for the targeted CpG site. Methylation data quality control, normalization, and filtering of redundant probes were conducted using the pipeline of the ChAMP. Differentially methylated probes (DMP) analysis was detected by the ‘champ.DMP’ function of ChAMP package.^41^ DMPs were considered for further analysis with a q value <0.05. The adjusted p value for multiple testing was calculated using the Benjamini-Hochberg correction.^31^

### Statistical analysis

The normality of the variables was tested by the Shapiro-Wilk normality test. For comparisons of two groups, statistical significance for normally distributed variables was estimated by an unpaired Student’s t-test, and non-normally distributed variables were analyzed by the Mann-Whitney U test. For comparisons of more than two groups, the Kruskal-Wallis and one-way analysis of variance tests were used for non-parametric and parametric methods, respectively. The correlation coefficient was computed by Spearman and distance correlation analyses. Χ^2^ test and two-sided Fisher’s exact tests were used to analyze contingency tables. The cut-off values of each data set were evaluated based on the association between survival outcome and signature score in each separate data set using the survminer package. The Kaplan-Meier method was used to generate survival curves for the subgroups in each data set, and the log-rank (Mantel-Cox) test was used to determine if they were statistically different. The HRs for univariate analyses were calculated using the univariate Cox proportional hazards regression model. The sensitivity and specificity of signature scores were depicted by the receiver operating characteristic (ROC) curve and quantified by the area under the ROC using the pROC package.^42^ The ‘roc.test’ function of pROC package was used to compare the area under the curve (AUC) or partial AUC of two correlated or uncorrelated ROC curves. All statistical analyses were conducted using R V.3.6.3.0 (https://www.r-project.org/), and the p values were two-sided. P values of less than 0.05 were considered statistically significant.

## Results

### TMEscore predicts ICB response of gastric cancer

**Figure 1:**
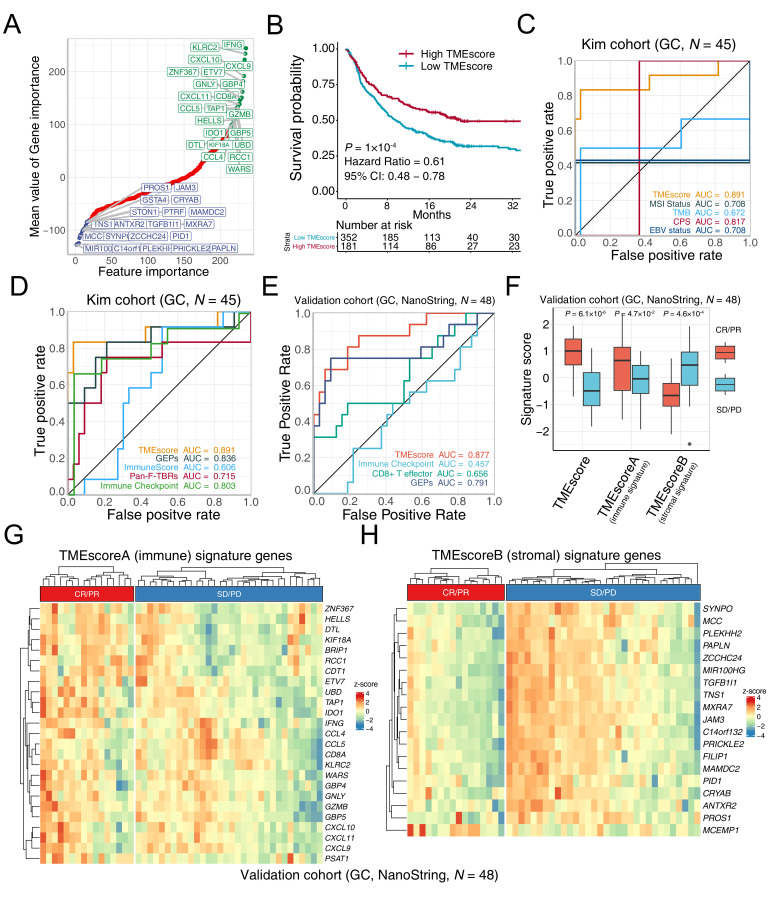
TMEscore holds promise in predicting immunotherapeutic response. (A) Feature engineering was conducted to minimize the number of TMEscore signature genes. Gene importance was exhibited with genes significantly associated with favorable immune checkpoint blockade (ICB) responses (top right, green), and genes correlated positively with immune exclusion and negatively with immunotherapeutic efficacy (bottom left, blue). (B) Kaplan-Meier survival analysis demonstrated that a high TMEscore was significantly related to more favorable overall survival in the study of multiple meta-data (p*=*1×10^−4^, HR=0.61, 95% CI: 0.48 to 0.78, cut-off=0.08). (C) Receiver operating characteristic (ROC) analyses indicated that the TMEscore harbored the highest area under the curve (AUC) (AUC=0.891) in comparison with other reported biomarkers of ICB, comprizing microsatellite instability (MSI) status, tumor mutation burden (TMB), programmed death-ligand 1 combined positive score (CPS), and Epstein-Barr virus (EBV) status in gastric cancer (AUC=0.708, 0.672, 0.817, 0.708, respectively; p values of pair comparison test see online supplemental table S7). (D) Tumor microenvironment (TME) relevant signatures and the TMEscore are estimated to compare the predictive sensitivity for responses. ROC analyses suggested that the TMEscore substantially outperformed these published transcriptomic-based methodologies for prediction of ICB treatment response, including gene expression profile scores (GEPs), ImmunoScore, pan-fibroblast transforming growth factor-beta (TGF-β) response signature (pan-fibroblast TGF-β response signature), and immune checkpoint (AUC=0.836, 0.606, 0.715, 0.803, respectively; detailed p values of pair comparison test see online supplemental table S7) (E–H) The predictive capacity of TMEscore for treatment response is corroborated in a multicenter clinical gastric cancer cohort. TMEscore possessed highest AUC surpassing immune checkpoint, CD8+ effector T cell and GEPs (AUC=0.877, 0.457, 0.656, 0.791, respectively); (E). Box plot supported that elevated TMEscore and TMEscoreA, as well as decreased TMEscoreB of responders (CR/PR) versus non-responder (SD/PD) in multicenter cohort (p=6.1×10^−6^, 4.7×10^−2^, 4.6×10^−4^, respectively); (F). Heatmaps exhibited the signature genes expression of TMEscoreA (G) and TMEscoreB (H), respectively, in the responsive (CR/PR) and the progressive (SD/PD) gastric cancer, validating prior results. CR, complete response; PD, progressive disease; PR, partial response; SD, stable disease.

To optimize the TME assessment for more efficient clinical translations, feature engineering (see online supplemental methods) was conducted in six ICB data sets (online supplemental table S1) and reduced TMEscore^15^ signature genes from 244 to 44. As previous research suggested,^15^ genes negatively associated with ICB response were enriched in immune exclusion phenotype (EMT/TGF-β pathway), whereas the immune relevant genes positively associated with treatment efficacy figure 1A, (online supplemental figure S1A). In several GC cohorts (online supplemental table S1), we found a consistent and closed association between the 44-gene TMEscore and the prior TMEscore measured of 244 genes (online supplemental figure S1B). Notably, the TMEscore was capable of serving as a prognostic biomarker of immunotherapy meta-cohort (GSE78220,^24^ IMvigor210,^6^ GSE93157,^43^ Snyder *et al*^44^ and TCGA-SKCM^25^) (figure 1B: TMEscore, p=0.0001; online supplemental figure S1C): TMEscoreA, p<0.0001 and online supplemental figure S1D: TMEscoreB, p=0.0396, respectively), and a predictive biomarker of ICB response in several independent cohorts (online supplemental figure S1E–L, online supplemental table S2. The AUCs of eight independent data sets indicated that the predictive value of simplifying TMEscroe (44 genes) was enhanced after dimension reduction (online supplemental figure S1E–L). In the advanced GC cohort receiving anti-PD-1 immunotherapy,^5^ the TMEscore yielded the highest AUC (AUC=0.891), surpassing other prevalent biomarkers, including MSI status, TMB, CPS and EBV infection (AUC=0.708, 0.672, 0.817, and 0.708, respectively) (figure 1C and online supplemental table S3), and several transcriptomic-based predictive counterparts, comprizing gene expression profile score (GEPs),^18^ ImmunoScore,^29^ CD8+ T effector score, and pan-fibroblast TGF-β response signature (Pan-F-TBRs)^6^(figure 1D).

10.1136/jitc-2021-002467.supp2 Supplementary data

We further measured expression of TMEscore genes in the tumor microenvironment, using NanoString nCounter platform^22^ and RNA isolated from tumor tissue obtained at baseline from 48 patients with advanced gastric cancer of multicenter before receiving ICB (table 1 and online supplemental table S4). Apparently, TMEscore achieves an overall accuracy of AUC=0.877, which is higher than other prevalent gene signature predictors^6 18 21^ and capturing almost all true responders (figure 1E, F). Consistent with our previous study,^15^ regressive tumors (complete response (CR)/partial response (PR)) were observed markedly higher TMEscoreA than stable and progressive tumors (progressive disease (PD)/stable disease (SD)), and TMEscoreB was negatively associated with the treatment efficacy of advanced GC (figure 1F, statistical p value of TMEscore, TMEscoreA and TMEscoreB were 6.1×10^−6^, 0.047 and 0.00046, respectively), implicating stromal activation as a critical mechanism of resistance to ICB.^6 15^ TMEscoreB (stromal-relevant) genes were more precise biomarker and significantly associated with treatment resistance, while TMEscoreA (immune-relevant) genes were highly expressed in a few non-responders (SD/PD) (figure 1G, H).

**Table 1:** Baseline characteristics of patients with advanced gastric cancer

	NanoString cohort	Kim cohort
	Total (n=48)	Total (n=61)
Age (years)	61.50 (27–76)	57 (26–78)
Sex		
	Male (28, 58%)	Male (43, 70%)
	Female (20, 42%)	Female (18, 30%)
Race		
	Asian (48, 100%)	Asian (61, 100%)
Type of specimens		
	FFPE (28, 58%)	FFPE (0, 0%)
	Biopsy (20, 42%)	Biopsy (61, 100%)
Clinical center		
	NFH (12, 25%)	Samsung Medical Center (61, 100%)
	SYSUCC (21, 44%)	
	GDPHCM (6, 13%)	
	TSAHSYSU (5, 10%)	
	TFAHSYSU (4, 8%)	
Type of checkpoint inhibitors		
	Camrelizumab (12, 25%)	Pembrolizumab (61, 100%)
	Toripalimab (12, 25%)	Camrelizumab (0, 0%)
	Sintilimab (11, 23%)	Toripalimab (0, 0%)
	Nivolumab (9, 19%)	Sintilimab (0, 0%)
	Pembrolizumab (4, 8%)	Nivolumab (0, 0%)
Regimen		
	Monotherapy (19, 40%)	Monotherapy (61, 100%)
	Combination (29, 60%)	Combination (0, 0%)
Number of previous therapies		
0	11 (23%)	0 (0%)
1	23 (48%)	32 (52.5%)
>=2	14 (29%)	29 (47.5%)

FFPE, formalin-fixed paraffin-embedded; GDPHCM, Guangdong Provincial Hospital of Chinese Medicine; NFH, Nanfang Hospital, Southern Medical University; SYSUCC, Sun Yat-sen University Cancer Center; TFAHSYSU, The First Affiliated Hospital of Sun Yat-sen University; TSAHSYSU, The Sixth Affiliated Hospital of Sun Yat-sen University.

### TMEscore predicts efficacy of checkpoint immunotherapy alone or combination with chemotherapy or angiogenesis inhibitor

**Figure 2:**
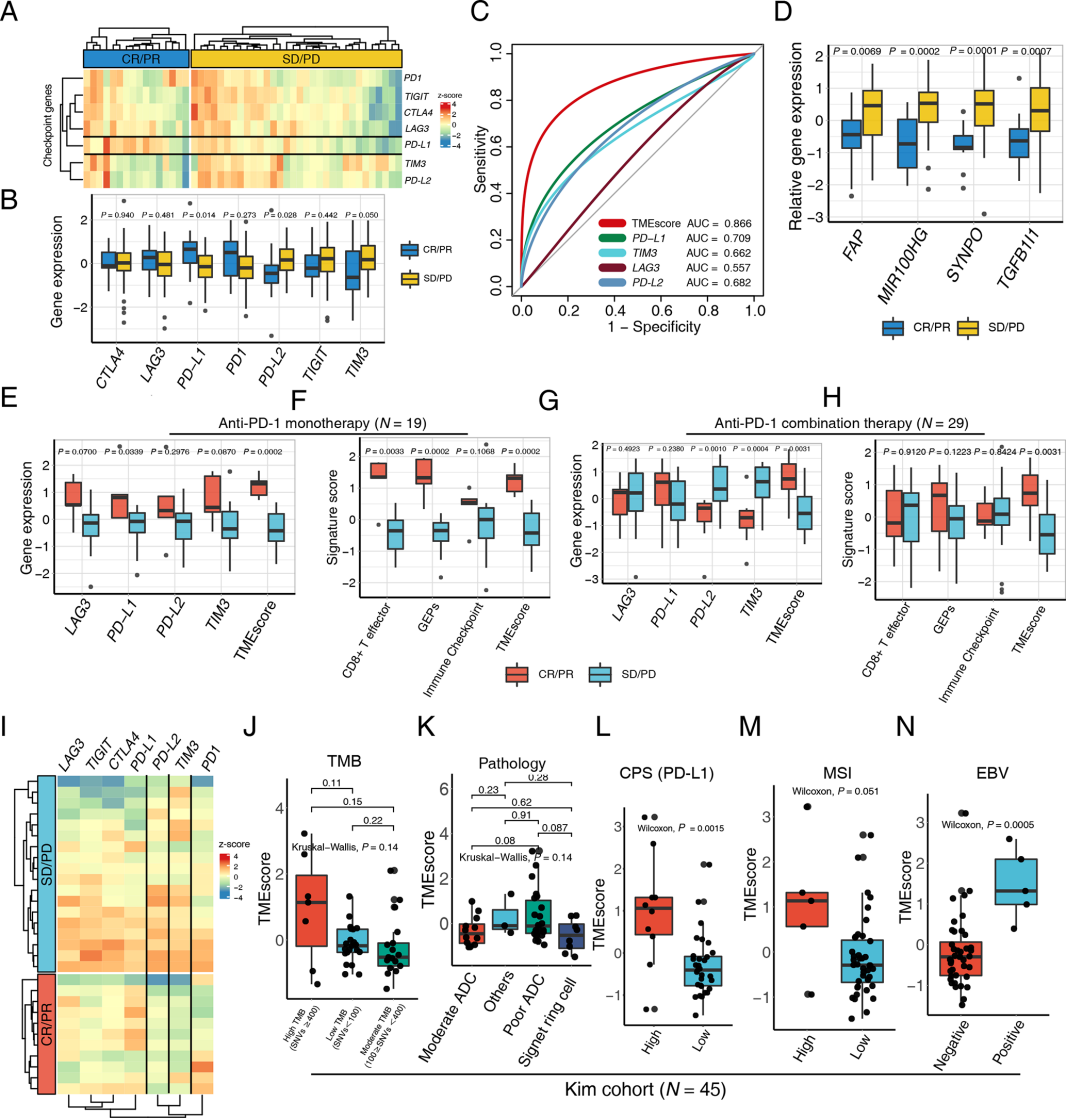
TMEscore predicts efficacy of checkpoint immunotherapy alone or combination with chemotherapy. (A) A heatmap numerated the expression of various immune checkpoint genes in the responder (blue) and the non-responder (yellow) subsets, highlighting upregulation of programmed death-ligand 1 (PD-L1) in responsive patients in a multicenter cohort of gastric cancer. (B) The box plot compared the expression levels of immune checkpoint genes in the responsive (blue) and non-responsive (yellow) cancer settings and corresponding p values were displayed on the top. (C) Receiver operating characteristic curve analysis demonstrated that the TMEscore with highest predictive efficacy for therapy sensitivity (area under the curve (AUC)=0.866), outperforming all the immune checkpoints comprizing *PD-L1*, *TIM3*, *LAG3*, and *PD-L2* (AUC=0.709, 0.662, 0.557, 0.682, respectively). (D) An elevation of stromal activation indexes, including *FAP, MIR100HG, SYNPO* and *TGFB1l1* (p=0.0069, 0.0002, 0.0001, 0.0007, respectively), was discovered in the patients with complete response (CR) or partial response (PR) relative to the counterparts. (E–H) An upregulation of the aforementioned immune checkpoints (E) and immunotherapy pertinent biomarkers (F) including TMEscore, was measured in the context of anti-programmed cell death protein 1 (PD-1) monotherapy, as well as anti-PD-1 combination therapy (G–H). Relevant p values were depicted on the top. (I) Heatmap demonstrated aforementioned immune checkpoint expression discrepancies in the setting of anti-PD-1 combination therapy responder (red) and non-responder (blue), indicative of the upregulation of *PD-L2* and *TIM3* in the non-responsive subset. (J) No statistical significance was observed between tumor mutation burden (TMB) and TMEscore (Kruskal-Wallis test, p=0.14). The number of non-synonymous single nucleotide variant ≥400 was defined as high mutational load (high TMB); 100–400, moderate mutation load (moderate TMB); and <100, low mutation load (low TMB). (K) A boxplot exhibited bare statistical significance in TMEscore diversity among different pathologies of gastric cancers (Kruskal-Wallis test, p=0.14). (L) An increase of TMEscore was observed in PD-L1 combined positive score (CPS) positive patients (Wilcoxon, p=0.0015). The specimen was considered to have high PD-L1 expression if CPS≥1. (M–N) A boxplot demonstrated that gastric cancers with high microsatellite instability (MSI) status (M) (Wilcoxon, p=0.051) and positive Epstein-Barr virus (EBV) infective status (N) (Wilcoxon, p=0.0005) harbored an elevated TMEscore. ADC, adenocarcinoma; PD, progressive disease; SD, stable disease.

To provide a precise map for understanding TMEscore performance in the context of mono- and combinational immunotherapy, we further explored the NanoString result of a 48 patients gastric cancer cohort. The expression of PD-L1 is prevailingly enriched in the responsive subset (CR/PR) relative to the progressive counterparts (figure 2A–C and online supplemental table S5). Intriguingly, the *PD-L2* and *TIM3* were significantly higher in non-responsive tumor, suggesting that upregulations of other corresponding or bypass checkpoint pathway may contribute to the resistance of PD-1 blockades (figure 2B–D and online supplemental table S5), by which according to reports the stromal activation and T-cell exclusion were induced.^6^ Additionally, *SYNPO* was reported to be upregulated during CAF activation,^45^ which is the critical mechanism of ICB resistance.

The clinical benefit of ICB monotherapy for advanced gastric cancer is limited, and recent clinical trials have demonstrated that combinations of ICBs with chemotherapy, anti-vascular targeted therapy or other molecular targeted therapies significantly improve treatment outcomes such as CheckMate-649.^46 47^ Consequently, there will be a pressing need for biomarkers that can be applied for patient selection for anti-PD-1 immunotherapy and chemotherapy combination. Among the multicenter data of GC, 19 patients received ICB monotherapy, and 29 patients were treated with ICBs combined with chemotherapy or other inhibitors (table 1). We systematically evaluated aforementioned biomarkers in both ICB monotherapy and the combination treatment settings. The majority of ICB relevant genes and immune relevant signatures were positively related to favorable mono-immunotherapy response, corroborating former discoveries (figure 2E, F and online supplemental figure S2A, B). Whereas their predictive efficacy significantly slid in therapy combination subset, especially the signatures related with immune activation (figure 2G, H). However, the TMEscore still harbored robust predictive capacities in both settings (figure 2G, H), possibly attributing to the superiorly essential influence exerted by stromal activation during synergic treatment (online supplemental figure S2C, D). Comparable trend of *PD-L2* and *TIM3* expression were also exhibited in the synergic therapy. Their upregulations in progressive patients suggested the potential pivotal molecular characteristics in shaping tumor immune evasion (figure 2G, I), which also implied the existence of synchronously upregulation of immune checkpoint pertinent genes, indicating this subset of patients may be latent candidate to benefit from *PD-L2* or *TIM3* pathway inhibitions.

### TMEscore accurately identifies more patients than MSI, EBV and TMB in mGC

In order to assess the predictive value and underlying mechanisms of TMEscore in advanced GC systematically, we performed integrative analysis across multi-omics data of advanced GC treated with pembrolizumab as a salvage treatment (NCT02589496, N=45) (online supplemental table S3), TCGA-STAD (N=375),^11^ and Asian Cancer Research Group (ACRG, N=299)^12^ cohorts (online supplemental table S6). A combination of the TMEscore with TMB or CPS (AUC=0.964, 0.973, respectively) observed a slight elevation in the AUC compared with TMEscore alone (AUC=0.921), despite no statistically significant discrepancy observed in pairwise comparisons (online supplemental figure S2E and online supplemental table S7). Intriguingly, the TMEscore was not correlated with tumor somatic mutation burden and histology subtypes in Kim cohort (figure 2J, K). However, in markedly stratified patients, when referring to levels of some biomarkers associated with ICB responsiveness,^5 48^ such as tumorous PD-L1 expression evaluated using CPS, MSI status and EBV infection, respectively (figure 2L–N). Accordingly, our analyses indicated that the TME estimation might have an alternative and more amenable mechanism than that of tumor intrinsic genomic features to serve as a robust biomarker for predicting ICB responses in advanced GC.

**Figure 3:**
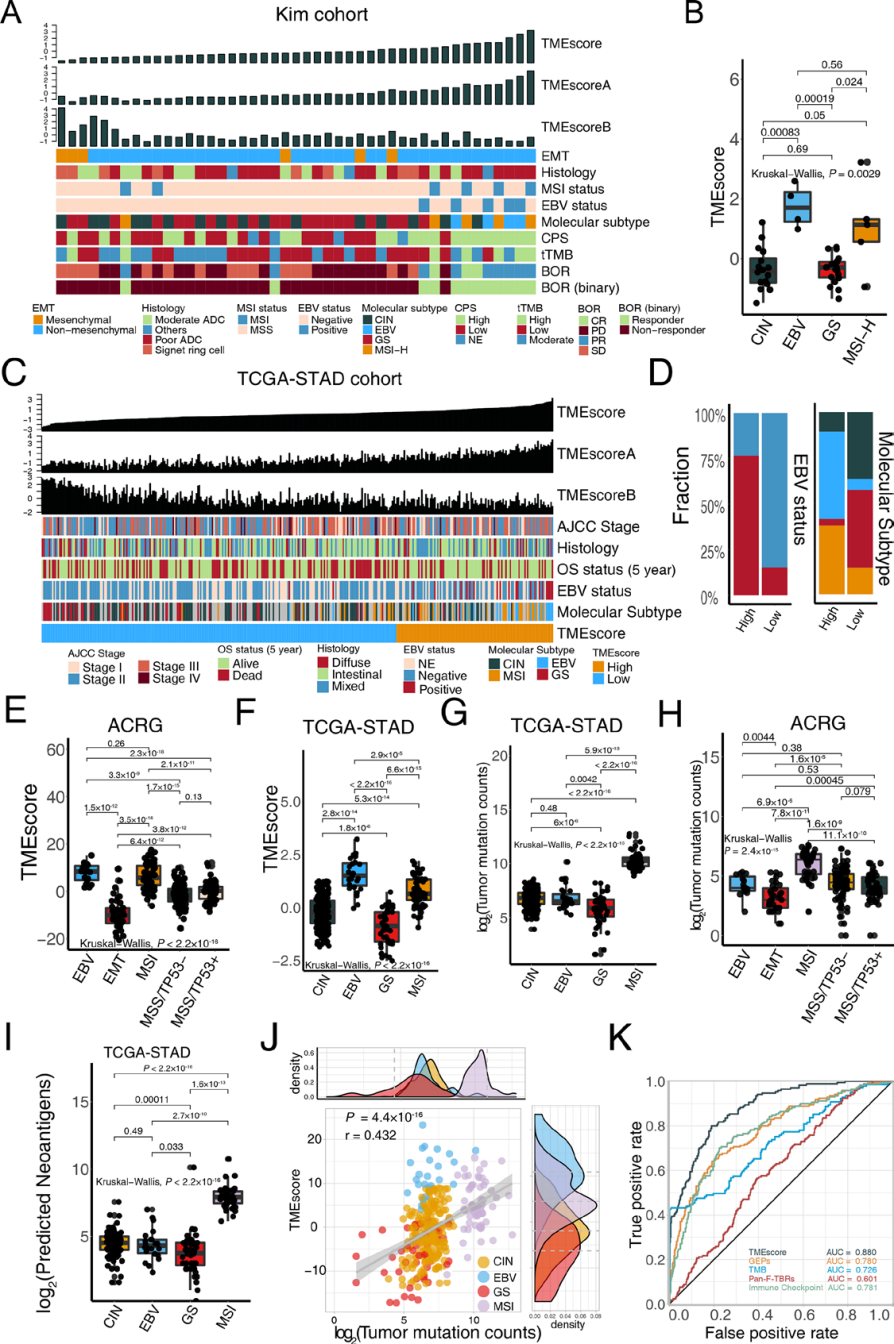
TMEscore is closely correlated with microsatellite instability-high (MSI-H) and Epstein-Barr virus (EBV) infective status in gastric cancer. (A) For each patient (columns) with metastatic gastric cancer, clinicopathological features and molecular characterizations were annotated. Column annotations represent epithelial–mesenchymal transition (EMT) (mesenchymal, non-mesenchymal); histology (moderate adenocarcinoma (ADC), poor ADC, signet ring cell, others); MSI status (MSS, MSI); EBV status (negative, positive); molecular subtype (chromosomal instability (CIN), EBV, genomically stable (GS), MSI-H); programmed death-ligand 1 combined positive score (CPS) (high, low, NE); tissue tumor mutation burden (tTMB); best overall response (BOR) (CR, PR, PD, SD); and binary BOR (responder, non-responder) for each sample. TMEscore, TMEscoreA, and TMEscoreB are displayed at the top of the panel. A high TMEscore is capable of identifying patients with EBV positive and MSI-H and responders to immune checkpoint blockade. (B) EBV and MSI gastric molecular subtype were substantially associated with higher TMEscore in the Kim cohort (Kruskal-Wallis test, p=0.0029). (C–D) For each patient (columns) in The Cancer Genome Atlas of Stomach Adenocarcinoma (TCGA-STAD) cohort, the landscape of clinicopathological features and molecular characterizations are displayed. Column annotations represent the AJCC stage (stage I, II, III, IV); OS 5-year (alive, dead); histology (diffuse, intestinal, mixed); EBV status (negative, positive, NE); molecular subtype (CIN, EBV, GS, MSI-H); and TME subtype (high, low) for each sample. TMEscore, TMEscoreA, and TMEscoreB are displayed at the top of the panel (C). Analysis of TCGA-STAD cohort corroborated that patients with EBV positive and MSI-H harbored a higher TMEscore (D) (Fisher’s exact test, p<2.2×10^−16^). (E–H) Boxplots indicated the TMEscore is substantially elevated in EBV and MSI molecular subtype either in both Asian Cancer Research Group (ACRG) (E) (Kruskal-Wallis test, p<2.2×10^−16^) and TCGA-STAD cohorts (F) (Kruskal-Wallis test, p<2.2×10^−16^). However, TMB is positively related to the MSI subtype but is not predictive of EBV status in both TCGA-STAD cohort (G) (Kruskal-Wallis test, p<2.2×10^−16^) and ACRG cohort (H) (Kruskal-Wallis test, p=2.4×10^−15^). (I) Neoantigens failed to identify EBV status in TCGA-STAD cohort, despite its significant correlation with MSI-H subtype (Kruskal-Wallis test, p<2.2×10^−16^). (J) A dotplot demonstrated a close correlation between TMB and the TMEscore. Every single dot represents one sample, corresponding molecular subtypes are identified in different colors (CIN: yellow, EBV: blue, GS: red, MSI: pink) (Spearman test, r=0.432, p=4.4×10^−16^). (K) ROC analyses suggested the TMEscore was predictive of EBV and MSI status of gastric cancer in TCGA-STAD and ACRG cohorts (n=634), with a higher AUC than that of gene expression profile scores and TMB (AUC=0.88, 0.78, 0.726, respectively). AJCC, The American Joint Committee on Cancer; OS, overall survival; CR, complete response; NE, unknown; PD, progressed disease; PR, partial response; SD, stable disease.

We depicted a landscape of the TME signature score, clinicopathological features, and molecular characterization in patients with metastatic GC treated with anti-PD-1 immunotherapy^5^ to investigate factors potentially associated with the treatment efficacy of ICB. We observed that patients with better responses were more likely to possess EBV and MSI-H molecular subtypes but were rarely enriched in chromosomal instability (CIN), genomically stable (GS), and EMT molecular subtypes (figure 3A, EBV and MSI-H: responders (n=9), non-responders (n=0); GS and CIN: responders (n=3), non-responders (n=33); p*=*2.5×10^−7^, Fisher’s exact test). Consistent with our recent research^15^ in TCGA-STAD and ACRG cohorts, the TMEscore was significantly higher in patients with MSI-H and EBV subtypes, relative to CIN and GS (figure 3B, p*=*0.003), suggesting that the predictiveness of the TMEscore was mostly contributed to molecular phenotype stratification. We next examined the predictive capacity of gene signatures and prevalent biomarkers in stratified patients with EBV and MSI-H molecular subtypes that indicates better responses to ICBs.^48 49^ ROC analyses indicated that the TMEscore (AUC=0.895) was superior in predicting EBV and MSI-H molecular subtypes, compared with MSI status, TMB, CPS, EBV status, GEPs, ImmuneScore, Pan-F-TBRs, and Immune Checkpoint (AUC=0.778, 0.781, 0.797, 0.708, 0.847, 0.646, 0.764, 0.767, respectively; online supplemental figure S2F–H and online supplemental table S7).

To validate above findings, we performed the same statistical analyses in two large multi-omics GC cohorts.^11 12^ We next focused on TCGA-STAD cohort^11^ and analyzed the clinical features (figure 3C and online supplemental figure S3A). In the low TMEscore group, the MSI and EBV subtypes were largely absent, while they took the majority of the group with the high TMEscore (EBV and MSI-H: high TMEscore (n=48), low TMEscore (n=16); GS and CIN: high TMEscore (n=25), low TMEscore (n=132); p<2.2×10^−16^, χ^2^ test; figure 3D). A similar trend was also observed in the ACRG cohort (EBV and MSI: high TMEscore (n=78), low TMEscore (n=8); other subtypes: high TMEscore (n=80), low TMEscore (n=134); p<2.2×10^−16^, χ^2^ test; Online supplemental figure S3B–D). Intriguingly, our analyses indicated that EBV infected tumors have comparable TMEscore with MSI-H tumors in the ACRG cohort (p*=*0.261; figure 3E) and even possessed a higher TMEscore than that of MSI-H tumors in TCGA cohort (p*=*2.9×10^−5^; figure 3F), whereas a significantly lower tumor mutation counts than that of MSI-H tumors in both TCGA-STAD and ACRG cohorts remained (p*=*5.9×10^−13^ and 6.9×10^−6^, respectively; figure 3G, H). We also noted a markedly lower neoantigen load in EBV infected tumors compared with MSI-H GC in TCGA cohort (p*=*2.7×10^−10^, figure 3I). Correlation analysis revealed that the TMEscore was positively associated with tumor mutation burden in both data sets (TCGA-STAD: p*=*4.4×10^−16^; figure 3J; ACRG: p*=*8.6×10^−11^; online supplemental figure S3D) and predicted neoantigen load in TCGA-STAD cohort (p*=*2.5×10^−11^; online supplemental figure S3E). Collectively, the EBV subtype remained at a low level of TMB and neoantigens with a high TMEscore and immune associated signatures in Pan-Caner cohorts (online supplemental figure S4A–G and online supplemental table S8). As shown by previous research^48 49^ about GC cohort treated with ICBs,^5^ patients with EBV infection, as well as the MSI-H phenotype, had an increased potential to benefit from ICB treatment. These observations further confirmed that TMB, as a widely used predictive biomarker,^50^ is incapable of identifying patients with GC with EBV subtype and tumor with virus infection (online supplemental figure 1 S5A-F), which also benefit from immunotherapy. As expected, the TMEscore could identify the EBV and MSI subtypes from all patients in TCGA-STAD and ACRG cohorts with significantly higher accuracy than TMB, GEPs,^18^ Pan-F-TBRs,^6^ and Immune checkpoint score^33^ (DeLong test, p*=*2.1×10^−6^, 8.8×10^−10^, 1.5×10^−32^, and 2.5×10^−8^, respectively; figure 3K and online supplemental table S7. Sufficiently, the aforementioned data confirmed that the TMEscore might perform better in selecting candidate patients with GC that can benefit from ICB immunotherapy.

### *ARID1A* and *PIK3CA* deficiency potentiate therapeutic antitumor immunity in gastric cancer

**Figure 4:**
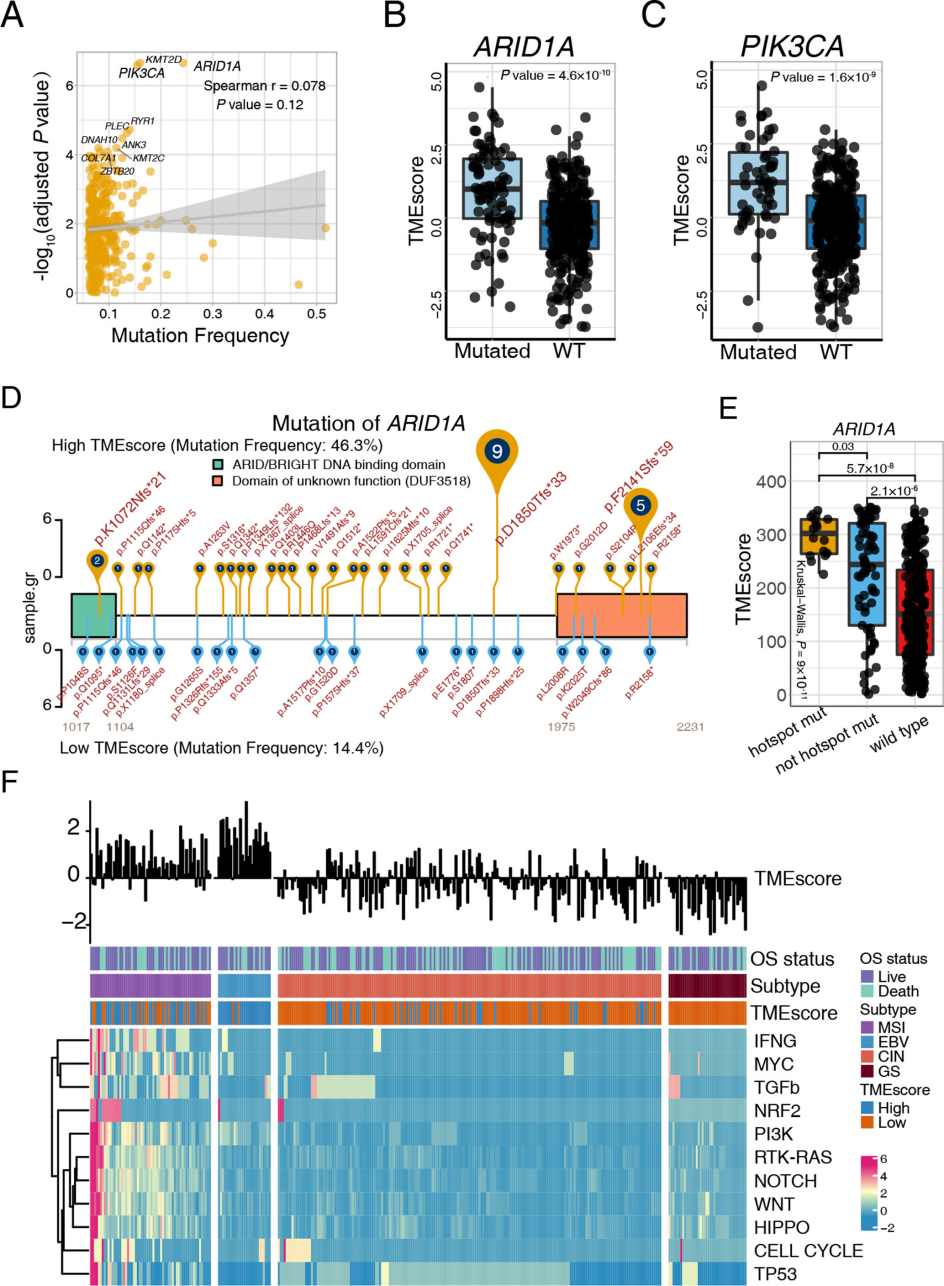
*ARID1A* and *PIK3CA* mutation potentiate antitumor immunity. (A) Mutation frequency and corresponding levels of TMEscores are exhibited in the dotplot, and the significance of *ARID1A*, *PIK3CA*, and *KMT2D* mutations are highlighted. Every single spot represents a gene, and statistical significance was shown through y-axis (Spearman test, r=0.078, p=0.12). (B–C) *ARID1A* (B) and *PIK3CA* (C) mutations were significantly associated with an increase of TMEscore. *ARID1A* (Wilcoxon, p=4.8×10^−10^) and *PIK3CA* (Wilcoxon, p=1.6×10^−9^) mutations were categorized in a binary way. (D–E) The landscape of the *ARID1A* mutation positions and corresponding TMEscore was displayed and highlighted p.D18550Tfs*33 and p.F2141Sfs*59 of *ARID1A* mutation in the high-TMEscore tumors. The mutation rates of high (yellow) and low (blue) TMEscores are shown (D). The *ARID1A* recurrent mutation is correlated with the higher TMEscore (Kruskal-Wallis test, p=9×10^−11^) (E). (F) The landscape of intrinsic pathway mutations (rows) is characterized for each sample (columns). Column annotations represent OS status (live, dead), molecular subtype (chromosomal instability (CIN), Epstein-Barr virus (EBV), genomic stable (GS) and microsatellite instability (MSI)); and tumor microenvironment (TME) subtype (high, low). The TMEscore is displayed in the top panel. Genomic mutations were limitedly enriched in the EBV molecular subtype, which exhibited a high TMEscore. Colors (blue to red) represent the corresponding expression levels (low to high). WT, wild type; OS, overall survival.

Somatic gene mutations can alter the vulnerability of cancer cells to T cells and T cell immunotherapies.^44 51 52^ We sought to uncover the immunogenomic determinants of therapeutic response and the tumor immune microenvironment activation of GC in two large patient cohorts (TCGA-STAD and ACRG). Mutations associated with TMEscore was identified utilizing Wilcoxon test and Fisher’s exact test (figure 4A and online supplemental table S9). Our analyses highlighted that mutation of *ARID1A* and *PIK3CA* (figure 4A), whether evaluated continuously (figure 4B, C) or binarily (online supplemental table S9), were markedly correlated with TMEscore levels in TCGA-STAD cohort, which were verified in the ACRG cohort (online supplemental figure S6A). Meanwhile, TMB was divided into high TMB group and low TMB group (cut-off=400, (online supplemental figure S6B) to analyze the relationship between TMEscore and *ARID1A* or *PIK3CA* mutations. As shown in online supplemental figure S6C, D, patients with *ARID1A* or *PIK3CA* mutations exhibited significantly higher TMEscore in the low TMB group. However, no significant trend was observed in the high TMB group. The above results suggested that in low TMB conditions, both *ARID1A* and *PIK3CA* mutations are associated with TME activation, while in high TMB conditions, the effect of *ARID1A* and *PIK3CA* mutations might be covered by the phenomenon that increasing neoantigens caused by abundant mutations further activating TME. *PIK3CA* is the most commonly mutated oncogene across all solid tumors.^53^ *ARID1A* deficiency, also a frequent mutation in various malignancies, has been reported to contribute to compromised mismatch repair (MMR), increased mutagenesis, and microsatellite instability genomic signature, and may cooperate with anti-PD-L1 therapy.^54^

Notably, we investigated further into the specific mutation locations to identify recurrent mutations with top mutation frequencies in binary TMEscore settings to visualize results by trackViewer.^55^ Intriguingly, p.D18550Tfs*33 and p.F2141Sfs*59 of the *ARID1A* mutation were highlighted in high-TMEscore tumors (figure 4D) and statistically correlated with TMEscore levels (p*=*0.03; figure 4E, online supplemental table S10). Gastric cancer with *PIK3CA* p.E545K and p.H1047R mutations were prominently enriched in the high-TMEscore group (online supplemental figure S7A, online supplemental table S10). However, limited statistical difference was observed in the continuous TMEscore despite the significant discrepancy across mutated and wild type (p*=*2.7×10^−8^; online supplemental figure S7B). Additionally, the mutation rate of *ARID1A and PIK3CA* in TCGA-STAD cohort were also higher in EBV and MSI molecular subtypes, which was correlated with an elevated TMEscore and immunotherapeutic response as compared with CIN and GS subtypes (*ARID1A:* p<2.2×10^−16^; *PIK3CA:* p<2.2×10^−16^; χ^2^ test; online supplemental figure S7C, D). We further found that the *ARID1A*-inactivating mutation in low TMB group was correlated with an upregulated immune checkpoint, CD8+ T effector, antigen presentation process (online supplemental figure S8A), and cellular response to glutamate metabolism (online supplemental figure S8B), which collectively suggested the higher T-cell infiltration and potential benefit from the blockade of ICB. Two recent studies indicated that the mutation of signaling pathways could serve as an immunotherapy biomarker^56^ and suggested combination therapy opportunities.^52^ The current study demonstrated pathway mutations derived predominantly from MSI molecular subtype (figure 4F and online supplemental table S11) and significant mutation accumulations of almost all pathways in the high-TMEscore fraction (figure 4F and online supplemental table S11). Nevertheless, in accordance with prior results (online supplemental figure S7D), a higher PI3K pathway mutation frequency was also observed in the EBV subtype in comparison with the GS and CIN subtypes, suggesting a latent interplay between EBV infections and the PI3K signaling pathway (online supplemental figure S9A and online supplemental table S11), which may partially explain the predominant increase of the TMEscore in EBV-infected patients (figure 3F and online supplemental figure S9B). Previous studies indicated that the interaction of *PIK3CA* mutation and EBV protein products may activate PI3K/ATK pathway which might be an initiator in tumorigenesis and progression. *PIK3CA* mutation revealed high intratumoral heterogeneity characterized with three to five different *PIK3CA* genotypes (including wildtype) in EBV-positive gastric cancer.^57^ Additionally, analyzing mutation signatures in the Catalog Of Somatic Mutations In Cancer^34^ indicated an intimate correlation between the TMEscore and mismatch repair associated signature 6 (online supplemental figure S9C and online supplemental table S12). Collectively, large data analyses of gastric TME elucidated the estimation of *ARID1A* and *PIK3CA* mutation status as a potential biomarker for immunotherapy strategies of GC.

### TME-associated metabolic characteristics

**Figure 5:**
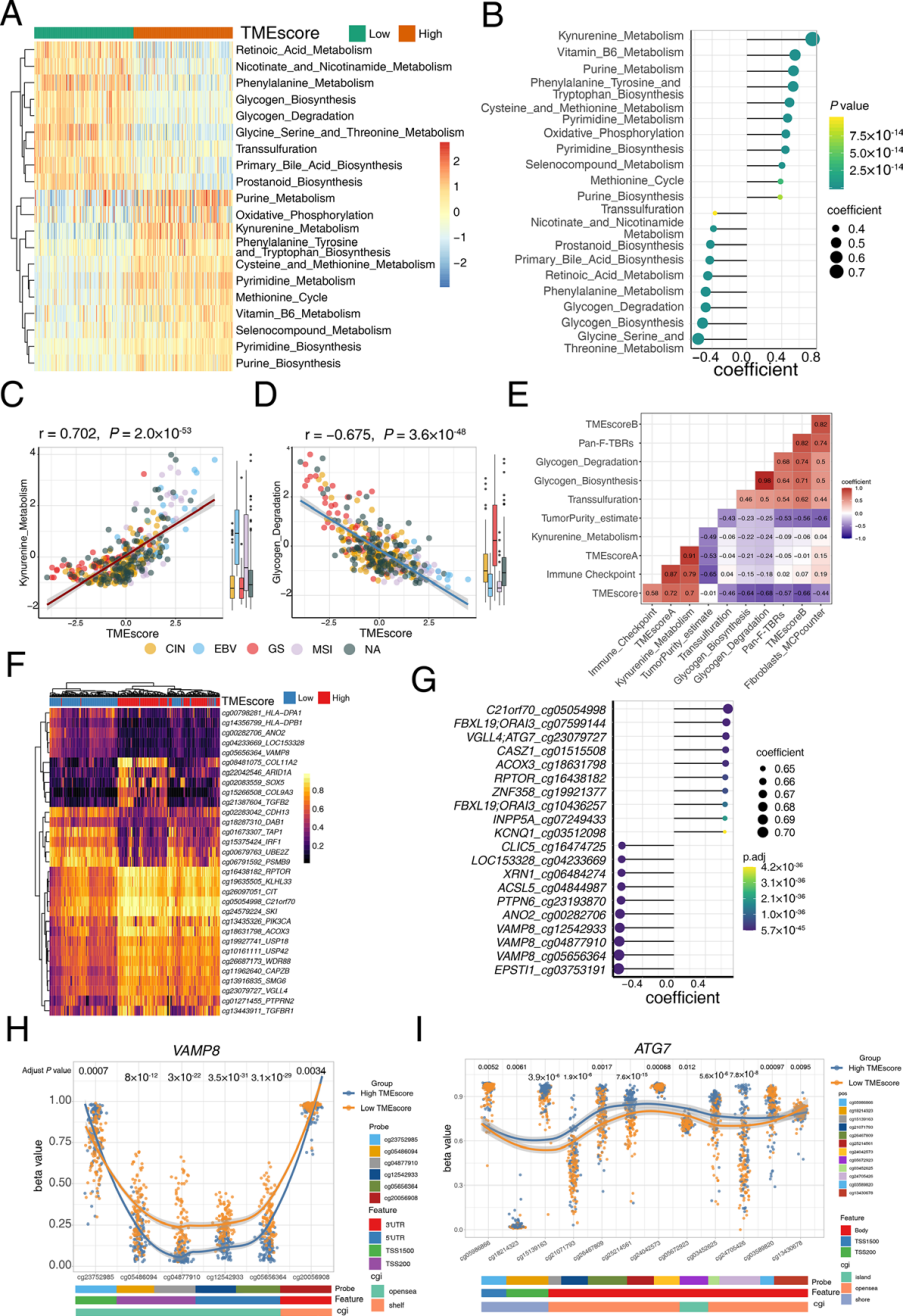
Tumor microenvironment (TME) associated metabolism and methylation characteristics. (A) Wilcoxon test show the differentially express metabolism pathway in high and low TMEscore tumor. For each patient (columns), signaling pathways (rows) are characterized in the heatmap. Colors (blue to red) represent the corresponding expression levels (low to high). Column annotations high (orange) and low (green) TMEscore. (B) Correlation analysis highlighted the most significant metabolism pathways in the high and low TMEscore tumors. Annotations of the pathways are listed on the left. Colors (yellow to green) represent p values, and the size of each dot represents the spearman coefficient. (C) A scatter plot demonstrated a close correlation between the TMEscore and kynurenine metabolism. Every single dot represents one sample, and corresponding molecular subtypes are identified in different colors (chromosomal instability (CIN): yellow, Epstein-Barr virus (EBV): blue, genomically stable (GS): red, microsatellite instability (MSI): pink; Spearman test, r=0.702, p=2.0×10^−53^). Kynurenine metabolism was significantly activated in EBV and MSI subtype (Kruskal-Wallis test, p=3.3×10^−10^). (D) A scatter plot demonstrated a close correlation between the TMEscore and glycogen metabolism. Every single dot represents one sample, and corresponding molecular subtypes are identified in different colors (CIN: yellow, EBV: blue, GS: red, MSI: pink; Spearman test, r=−0.675, p=3.6×10^−48^). Glycogen metabolism was significantly activated in GS subtype (Kruskal-Wallis test, p<2.2×10^−16^). (E) A corrplot displays correlations among kynurenine metabolism, glycogen metabolism and TME-related signatures. Coefficients are characterized in number. Colors red and purple represent positive and negative correlations. (F) The heatmap exhibited the landscape of differentially methylated genes in high and low TMEscore tumors. For each patient (columns), significant methylated regions of specific genes (rows, annotated on the right) are characterized. The column annotations represent high (red) and low (blue) TMEscore. Colors (yellow to purple) represent the corresponding methylation levels (low to high). (G) Correlation analysis highlighted the top 20 methylated probes and genes in the high and low TMEscore tumors. Annotations of the probes and genes are listed on the left. Colors (green to purple) represent p values, and the size of each dot represents the spearman coefficient. (H) The discrepancy of *VAMP8* methylation in different regions in high (blue) and low (yellow) TMEscore. Annotations of probes (cg23752985, cg05486094, cg04877910, cg12542933, cg05656364, cg20056908), features (3′UTR, 5′UTR, TSS1500, TSS200), and CpG islands (CGI) (opensea, shelf) are exhibited on the bottom panel. (I) The discrepancy of *ATG7* methylation in different regions in high (blue) and low (yellow) TMEscore. Pan-F-TBRs, pan-fibroblast TGF-β response signature.

Given the intriguing metabolic regulations observed in the different *ARID1A-*mutant statuses, we further explored transcriptomic profiles and dissected the latent intrinsic mechanism contributing to the crucial predictive capacity of the TMEscore. Metabolic signatures were estimated by PCA methodology^6^ and comprehensively investigated in TCGA-STAD cohort. Correlation analysis highlighted that kynurenine metabolism, purine metabolism and cysteine metabolism were activated in the high-TMEscore subset, while glycogen metabolism, transsulfuration, and glycine serine metabolism were significantly upregulated in low TMEscore group (figure 5A, B). Statistical analysis suggested that kynurenine metabolism was closely correlated with a high TMEscore (p=2.0×10^−53^, r=0.702; figure 5C and online supplemental table S13) and immunotherapy-favorable molecular subtypes including EBV and MSI-H (Kruskal-Wallis, p=3.3×10^−10^; figure 5C). The downregulated kynurenine metabolism was also observed to suggest T cell exclusion, which may indicate insensitivity to ICB therapy (figure 5E). Kynurenine metabolism processes may be a promising target to restore tumor-restraining T-cell immunogenicity and therefore promote ICB therapeutic efficacy in gastric cancer, such as IDO1 inhibitor.^58^ We observed that glycogen metabolism was significantly activated in low TMEscore tumors and immune exclusive molecular subtypes both in TCGA-STAD cohort and ACRG cohort (figure 5D and online supplemental figure S10A–F), which suggest that it may be correlated with immune exclusion phenotype (figure 5E and online supplemental figure S10G, H) and mediate treatment resistance of immunotherapy. Consistently, Curtis *et al* indicated that the interaction between cancer cells and CAFs supported glycogenolysis which funneled into glycolysis, leading to increased proliferation, immune evasion, and metastasis of cancer cells.^59^ Together, we identified a collection of metabolism characteristics and biological processes associated with TME, which reflects the intricacy of the TME and indicates potential combination therapy opportunities.

### Methylation regions correlate with immune activity

A prior study^60^ demonstrated that a high m6Ascore indicates an immune-exclusion TME phenotype, stromal activation, decreased survival, decreased neoantigen load, and inferior response in GC. Thereafter, we attempted to identify the epigenetic immunomodulation involved in the antitumor immunity and tumor immune editing, which may be fundamental for understanding the inflammatory reaction that occurs in the diseases. Notably, a comprehensive investigation into the DNA methylation position landscape suggested demethylation of the *VAMP8*, was enriched in the low-TMEscore cluster, with the demethylation of the *ATG7* in the high-TMEscore cluster (figure 5F–I and online supplemental table S14). Intriguingly, further exploration of corresponding methylation regions revealed that cg04877910, cg12542933, cg05656364, cg05486094 and cg20056908 of *VAMP8* methylation were consistently negatively associated with high TMEscore and MSI and EBV molecular subtypes, whereas cg23752985 of *VAMP8* methylation harbored a relatively diverse distribution in molecular subtypes and correlations with the TMEscore (online supplemental figure S11A, B). Enrichment of differentially methylated genes highlighted the vital role *VAMP8* methylation plays in the TME regulatory network via upregulating immune pathways, comprizing pathways of leukocyte activation regulation, protein location to the membrane, antigen processing and presentation, coated vesicle, and recycling endosome (online supplemental figure S11C), which indicated the crucial role *VAMP8* plays in the complex gene interactions and crosstalk in extensive signaling pathways. Additionally, the demethylation of *ATG7,* as a gene marker of autophagy, is significantly correlated TMEscore (online supplemental figure S11D). Further analyses of the relationship among discovered *ATG7*-associated signatures (positive regulation of autophagy) indicated that demethylation of the *ATG7* was contributed to the immune exclusion in TME, with elevated TMEscoreB and fibroblast infiltration in TCGA-STAD and ACRG cohort (online supplemental figure S11E, F). Collectively, DNA methylation, such as different methylation regions of *VAMP8* and *ATG7*, may offer a lens into the complexity and diversity of the TME and immune-activity determination, thereafter might assist in optimizing immunotherapy strategies.

## Discussion

Our studies leveraging multi-omics data highlight TME evaluation (TMEscore) as a predictor of tumor immunogenicity and objective response rates and overall survival in six independent cohorts treated with ICBs. Moreover, the synergic therapy of ICB with chemotherapy or angiogenesis inhibitor is encountering the dilemmas of lacking functional molecular biomarkers. Notably, based on a multicenter clinical gastric cancer cohort, we discovered TMEscore is robust in predicting treatment efficacy in the context of checkpoint immunotherapy alone or its combination with chemotherapy or angiogenesis inhibitor, where the predictive accuracy of immune activation relevant signatures markedly shrinks.

Given the promising predictive value of TMEscore, we systematically investigated TMEscore pertinent underlying mechanisms to reinforce our refined understanding of the interplay between tumor-intrinsic features and TME and offer novel precise methodologies to accelerate precision immunotherapy. Selection strategies of optimal biomarkers remain controversial due to complicated clinical applications.^9 10^ For example, though PD-L1 expression level indicated therapeutic benefit, patients with PD-L1 <1% also responded to ICBs.^1^ In current study, the TMEscore substantially outperformed the counterparts including PD-L1 abundance, TMB, and MSI-H in discriminating response to ICBs.^9 10^ Merits of TMEscore is mainly attributed to the accurate identification of immune microenvironment activation, especially high CD8+ T cell infiltration tumors, immune exclusion and EBV infection status. Notably, EBV infection commonly accompanies with a low TMB, but is a unique marker with a high potential for response to ICB in GC,^5 48^ which was consistently confirmed by Subudhi *et al* in the setting of prostate cancer.^61^

Although TMB is a wide-recommended biomarker, specific alterations usually initiate carcinogenesis and neo-antigens generation but their roles in immune therapy sensitivity remain obscure. We identified mutations of *ARID1A* and *PIK3CA* associated with immune activation facilitating checkpoint immunotherapy. *ARID1A* is a component of the SWI/SNF chromatin remodeling complex,^62^ frequently mutated in GC.^11 12^ *ARID1A* deficiency closely correlates with the ICB response,^54^ potentially attributing to impairing MMR and elevating PD-L1 expression. Our study unprecedently proposed that *ARID1A* deficiency reformed the TME, with two specific *ARID1A* mutation locations of p.D18550Tfs*33 and p.F2141Sfs*59 harboring markedly higher TMEscore. Current work also indicated a potential interaction between *ARID1A*^48^ and *PIK3CA*^11^ mutations and EBV infection, partially explaining the elevated TMEscore in EBV subtype.

Metabolically speaking, we discovered that activation of kynurenine metabolism was correlated with EBV infection and MSI-H status subsequently upregulate immune suppressive markers, such as PD-L1 and IDO. Consistently, a recent report indicated the mechanistic link between kynurenine metabolism and the immunosuppressive microenvironment.^63 64^ Therefore, the inhibition of kynurenine metabolism may be a potential target for combinational therapy to improve the efficacy of ICB.^58^

DNA methylation guided the epigenetic regulation of genes, which was not limited in cancer cells but also immune cells and stromal cells, thereafter hypomethylation of specific genes could modify TME components and their interactions.^65^ Xiao *et al* have emphasized the contribution of the specific gene *SOCS1* methylation of CAFs made in reprogramming the TME induced by PDAC cells.^66^ Similarly, our analysis of DNA methylation landscape highlighted another gene methylation, *VAMP8*, correlated with the TME and immune-activity-related pathways. Additionally, extensive exploration of different methylation regions of *VAMP8* exhibited an inverse trend in different TMEscore groups, thereby offering a novel understanding of complex interplay linking methylation with TME. Macroautophagy is an essential cellular catabolic process required for survival under conditions of starvation. Recent study indicated that loss of *ATG7* in cancer cells which mediates autophagy disruption can enhance antitumor immune responses.^67^ Our data suggest that *ATG7* demethylation was closely associated with immune exclusion and CAF infiltration, which may provide insights into possible mechanisms.

Despite the TMEscore presenting high sensitivity in predicting immunotherapy efficacy, its application may be limited across diverse cancer types.^15^ Tumor heterogeneity and tissue specificity are presumed to be the main reasons and could also be interpreted by the various immune microenvironments. We are collecting a large number of gastric cancer samples before immunotherapy to determine an appropriate TMEscore cut-off value for consequent clinical practice. To develop TMEscore into a clinical-grade immunotherapy biomarker, we are devoted to carrying out two clinical trial of gastric cancer treated with ICBs (NCT04850716, NCT04850729).

## Conclusions

Collectively, we optimized a TME evaluation tool that may serve as a robust biomarker and integrated it as an open-source R package for further application in clinical implementation. The predictive capacity of TMEscore was verified in two advanced gastric cancer cohorts, which highlighted the predictive efficacy of tumor microenvironment evaluation. The intrinsic features involving the *ARID1A* and *PIK3CA* mutations, kynurenine metabolism, glycogen metabolism, *ATG7* and *VAMP8* methylation provide new insight into the potential mechanisms of TMEscore-guided precision immunotherapies.

## Data Availability

Data are available in a public, open access repository. Data are available upon reasonable request. Data may be obtained from a third party and are not publicly available. The raw sequencing data have been deposited at the European Nucleotide Archive and are available under accession number RJEB25780. The analytic code and package used to estimate the TMEscore and prevalent signature are provided for non-commercial use at GitHub: https://github.com/DongqiangZeng0808/TMEscore and https://github.com/IOBR/IOBR. A detailed README file is also available, complete with examples of how to use the package.

## References

[1] Kang Y-K, Boku N, Satoh T, et al. Nivolumab in patients with advanced gastric or gastro-oesophageal junction cancer refractory to, or intolerant of, at least two previous chemotherapy regimens (ONO-4538-12, ATTRACTION-2): a randomised, double-blind, placebo-controlled, phase 3 trial. Lancet 2017;390:2461–71. 10.1016/S0140-6736(17)31827-5 28993052

[2] Fuchs CS, Doi T, Jang RW, et al. Safety and efficacy of pembrolizumab monotherapy in patients with previously treated advanced gastric and gastroesophageal junction cancer: phase 2 clinical KEYNOTE-059 trial. JAMA Oncol 2018;4:e180013. 10.1001/jamaoncol.2018.0013 29543932 PMC5885175

[3] Shitara K, Özgüroğlu M, Bang Y-J, et al. Pembrolizumab versus paclitaxel for previously treated, advanced gastric or gastro-oesophageal junction cancer (KEYNOTE-061): a randomised, open-label, controlled, phase 3 trial. Lancet 2018;392:123–33. 10.1016/S0140-6736(18)31257-1 29880231

[4] Nanda R, Chow LQM, Dees EC, et al. Pembrolizumab in patients with advanced triple-negative breast cancer: phase Ib KEYNOTE-012 study. J Clin Oncol 2016;34:2460–7. 10.1200/JCO.2015.64.8931 27138582 PMC6816000

[5] Kim ST, Cristescu R, Bass AJ, et al. Comprehensive molecular characterization of clinical responses to PD-1 inhibition in metastatic gastric cancer. Nat Med 2018;24:1449–58. 10.1038/s41591-018-0101-z 30013197

[6] Mariathasan S, Turley SJ, Nickles D, et al. Tgfβ attenuates tumour response to PD-L1 blockade by contributing to exclusion of T cells. Nature 2018;554:544–8. 10.1038/nature25501 29443960 PMC6028240

[7] Tunger A, Sommer U, Wehner R, et al. The evolving landscape of biomarkers for anti-PD-1 or anti-PD-L1 therapy. J Clin Med 2019;8. 10.3390/jcm8101534. [Epub ahead of print: 25 Sep 2019]. 31557787 PMC6832659

[8] Meléndez B, Van Campenhout C, Rorive S, et al. Methods of measurement for tumor mutational burden in tumor tissue. Transl Lung Cancer Res 2018;7:661–7. 10.21037/tlcr.2018.08.02 30505710 PMC6249625

[9] Addeo A, Banna GL, Weiss GJ. Tumor mutation burden-from hopes to doubts. JAMA Oncol 2019;5:934–5. 10.1001/jamaoncol.2019.0626 31145420

[10] Wood MA, Weeder BR, David JK, et al. Burden of tumor mutations, neoepitopes, and other variants are weak predictors of cancer immunotherapy response and overall survival. Genome Med 2020;12:33. 10.1186/s13073-020-00729-2 32228719 PMC7106909

[11] Cancer Genome Atlas Research Network. Comprehensive molecular characterization of gastric adenocarcinoma. Nature 2014;513:202–9. 10.1038/nature13480 25079317 PMC4170219

[12] Cristescu R, Lee J, Nebozhyn M, et al. Molecular analysis of gastric cancer identifies subtypes associated with distinct clinical outcomes. Nat Med 2015;21:449–56. 10.1038/nm.3850 25894828

[13] Bruni D, Angell HK, Galon J. The immune contexture and immunoscore in cancer prognosis and therapeutic efficacy. Nat Rev Cancer 2020;20:662–80. 10.1038/s41568-020-0285-7 32753728

[14] Zeng D, Zhou R, Yu Y, et al. Gene expression profiles for a prognostic immunoscore in gastric cancer. Br J Surg 2018;105:1338-1348. 10.1002/bjs.10871 29691839 PMC6099214

[15] Zeng D, Li M, Zhou R, et al. Tumor microenvironment characterization in gastric cancer identifies prognostic and Immunotherapeutically relevant gene signatures. Cancer Immunol Res 2019;7:737–50. 10.1158/2326-6066.CIR-18-0436 30842092

[16] Zeng D, Ye Z, Wu J, et al. Macrophage correlates with immunophenotype and predicts anti-PD-L1 response of urothelial cancer. Theranostics 2020;10:7002–14. 10.7150/thno.46176 32550918 PMC7295060

[17] Fridman WH, Zitvogel L, Sautès-Fridman C, et al. The immune contexture in cancer prognosis and treatment. Nat Rev Clin Oncol 2017;14:717–34 https://www.nature.com/articles/nrclinonc.2017.101#supplementary-information 10.1038/nrclinonc.2017.101 28741618

[18] Cristescu R, Mogg R, Ayers M, et al. Pan-tumor genomic biomarkers for PD-1 checkpoint blockade-based immunotherapy. Science 2018;362:6411. 10.1126/science.aar3593 PMC6718162 30309915

[19] Koliaraki V, Prados A, Armaka M, et al. The mesenchymal context in inflammation, immunity and cancer. Nat Immunol 2020;21:974–82. 10.1038/s41590-020-0741-2 32747813

[20] Derynck R, Turley SJ, Akhurst RJ. Tgfβ biology in cancer progression and immunotherapy. Nat Rev Clin Oncol 2021;18:9–34. 10.1038/s41571-020-0403-1 32710082 PMC9721352

[21] Ayers M, Lunceford J, Nebozhyn M, et al. IFN-γ-related mRNA profile predicts clinical response to PD-1 blockade. J Clin Invest 2017;127:2930–40. 10.1172/JCI91190 28650338 PMC5531419

[22] Geiss GK, Bumgarner RE, Birditt B, et al. Direct multiplexed measurement of gene expression with color-coded probe pairs. Nat Biotechnol 2008;26:317–25. 10.1038/nbt1385 18278033

[23] Ulloa-Montoya F, Louahed J, Dizier B, et al. Predictive gene signature in MAGE-A3 antigen-specific cancer immunotherapy. J Clin Oncol 2013;31:2388–95. 10.1200/JCO.2012.44.3762 23715562

[24] Hugo W, Zaretsky JM, Sun L, et al. Genomic and transcriptomic features of response to anti-PD-1 therapy in metastatic melanoma. Cell 2016;165:35–44. 10.1016/j.cell.2016.02.065 26997480 PMC4808437

[25] Cancer Genome Atlas Network. Genomic classification of cutaneous melanoma. Cell 2015;161:1681–96. 10.1016/j.cell.2015.05.044 26091043 PMC4580370

[26] Auslander N, Zhang G, Lee JS, et al. Robust prediction of response to immune checkpoint blockade therapy in metastatic melanoma. Nat Med 2018;24:1545–9. 10.1038/s41591-018-0157-9 30127394 PMC6693632

[27] Lesterhuis WJ, Rinaldi C, Jones A, et al. Network analysis of immunotherapy-induced regressing tumours identifies novel synergistic drug combinations. Sci Rep 2015;5:12298. 10.1038/srep12298 26193793 PMC4508665

[28] Zeng D, Ye Z, Shen R. IOBR: multi-omics Immuno-oncology biological research to decode tumor microenvironment and signatures. Frontiers in Immunol 2020;12:2547. 10.3389/fimmu.2021.687975 PMC8283787 34276676

[29] Yoshihara K, Shahmoradgoli M, Martínez E, et al. Inferring tumour purity and stromal and immune cell admixture from expression data. Nat Commun 2013;4:2612. 10.1038/ncomms3612 24113773 PMC3826632

[30] Love MI, Huber W, Anders S. Moderated estimation of fold change and dispersion for RNA-Seq data with DESeq2. Genome Biol 2014:1474–760. 10.1186/s13059-014-0550-8 PMC4302049 25516281

[31] Benjamini Y, Hochberg Y. Controlling the false discovery rate: a practical and powerful approach to multiple testing. J R Stat Soc Ser B 1995;57:289–300. 10.1111/j.2517-6161.1995.tb02031.x

[32] Colaprico A, Silva TC, Olsen C, et al. TCGAbiolinks: an R/Bioconductor package for integrative analysis of TCGA data. Nucleic Acids Res 2016;44:e71. 10.1093/nar/gkv1507 26704973 PMC4856967

[33] Rosenthal R, McGranahan N, Herrero J, et al. DeconstructSigs: delineating mutational processes in single tumors distinguishes DNA repair deficiencies and patterns of carcinoma evolution. Genome Biol 2016;17:31. 10.1186/s13059-016-0893-4 26899170 PMC4762164

[34] Sondka Z, Bamford S, Cole CG, et al. The cosmic cancer gene census: describing genetic dysfunction across all human cancers. Nat Rev Cancer 2018;18:696–705. 10.1038/s41568-018-0060-1 30293088 PMC6450507

[35] Yu G, Wang L-G, Han Y, et al. clusterProfiler: an R package for comparing biological themes among gene clusters. OMICS 2012;16:284–7. 10.1089/omi.2011.0118 22455463 PMC3339379

[36] Subramanian A, Tamayo P, Mootha VK, et al. Gene set enrichment analysis: a knowledge-based approach for interpreting genome-wide expression profiles. Proc Natl Acad Sci U S A 2005;102:15545–50. 10.1073/pnas.0506580102 16199517 PMC1239896

[37] Hänzelmann S, Castelo R, Guinney J. GSVA: gene set variation analysis for microarray and RNA-Seq data. BMC Bioinformatics 2013;14:7. 10.1186/1471-2105-14-7 23323831 PMC3618321

[38] The gene ontology consortium. The gene ontology resource: 20 years and still going strong. Nucleic Acids Res 2018;47:D330–8. 10.1093/nar/gky1055 PMC6323945 30395331

[39] Kanehisa M, Sato Y, Kawashima M, et al. Kegg as a reference resource for gene and protein annotation. Nucleic Acids Res 2016;44:D457–62. 10.1093/nar/gkv1070 26476454 PMC4702792

[40] Liberzon A, Birger C, Thorvaldsdóttir H, et al. The molecular signatures database (MSigDB) hallmark gene set collection. Cell Syst 2015;1:417–25. 10.1016/j.cels.2015.12.004 26771021 PMC4707969

[41] Morris TJ, Butcher LM, Feber A, et al. Champ: 450k CHIP analysis methylation pipeline. Bioinformatics 2014;30:428–30. 10.1093/bioinformatics/btt684 24336642 PMC3904520

[42] Robin X, Turck N, Hainard A, et al. pROC: an open-source package for R and S+ to analyze and compare ROC curves. BMC Bioinformatics 2011;12:77. 10.1186/1471-2105-12-77 21414208 PMC3068975

[43] Prat A, Navarro A, Paré L, et al. Immune-Related gene expression profiling after PD-1 blockade in non-small cell lung carcinoma, head and neck squamous cell carcinoma, and melanoma. Cancer Res 2017;77:3540–50. 10.1158/0008-5472.CAN-16-3556 28487385

[44] Anagnostou V, Smith KN, Forde PM, et al. Evolution of neoantigen landscape during immune checkpoint blockade in non-small cell lung cancer. Cancer Discov 2017;7:264–76. 10.1158/2159-8290.CD-16-0828 28031159 PMC5733805

[45] Lee YT, Tan YJ, Falasca M, et al. Cancer-Associated fibroblasts: epigenetic regulation and therapeutic intervention in breast cancer. Cancers 2020;12. 10.3390/cancers12102949. [Epub ahead of print: 13 Oct 2020]. PMC7600259 33066013

[46] Moehler MH, Janjigian YY, Adenis A, et al. CheckMate 649: a randomized, multicenter, open-label, phase III study of nivolumab (NIVO) + ipilimumab (IPI) or nivo + chemotherapy (CTX) versus CTX alone in patients with previously untreated advanced (AdV) gastric (G) or gastroesophageal junction (GEJ) cancer. JCO 2018;36:TPS192. 10.1200/JCO.2018.36.4_suppl.TPS192

[47] Moehler M, Shitara K, Garrido M, et al. LBA6_PR Nivolumab (nivo) plus chemotherapy (chemo) versus chemo as first-line (1L) treatment for advanced gastric cancer/gastroesophageal junction cancer (GC/GEJC)/esophageal adenocarcinoma (EAC): first results of the CheckMate 649 study. Ann Oncol 2020;31:S1191. 10.1016/j.annonc.2020.08.2296

[48] Panda A, Mehnert JM, Hirshfield KM, et al. Immune activation and benefit from Avelumab in EBV-positive gastric cancer. J Natl Cancer Inst 2018;110:316–20. 10.1093/jnci/djx213 29155997 PMC6658862

[49] Le DT, Uram JN, Wang H, et al. Pd-1 blockade in tumors with mismatch-repair deficiency. N Engl J Med 2015;372:2509–20. 10.1056/NEJMoa1500596 26028255 PMC4481136

[50] Samstein RM, Lee C-H, Shoushtari AN, et al. Tumor mutational load predicts survival after immunotherapy across multiple cancer types. Nat Genet 2019;51:202–6. 10.1038/s41588-018-0312-8 30643254 PMC6365097

[51] Braun DA, Hou Y, Bakouny Z, et al. Interplay of somatic alterations and immune infiltration modulates response to PD-1 blockade in advanced clear cell renal cell carcinoma. Nat Med 2020;26:909–18. 10.1038/s41591-020-0839-y 32472114 PMC7499153

[52] Benci JL, Johnson LR, Choa R, et al. Opposing functions of interferon coordinate adaptive and innate immune responses to cancer immune checkpoint blockade. Cell 2019;178:e14:933–48. 10.1016/j.cell.2019.07.019 PMC6830508 31398344

[53] Bailey MH, Tokheim C, Porta-Pardo E, et al. Comprehensive characterization of cancer driver genes and mutations. Cell 2018;173:e18:371–85. 10.1016/j.cell.2018.02.060 PMC6029450 29625053

[54] Shen J, Ju Z, Zhao W, et al. ARID1A deficiency promotes mutability and potentiates therapeutic antitumor immunity unleashed by immune checkpoint blockade. Nat Med 2018;24:556–62. 10.1038/s41591-018-0012-z 29736026 PMC6076433

[55] Ou J, Zhu LJ. trackViewer: a Bioconductor package for interactive and integrative visualization of multi-omics data. Nat Methods 2019;16:453–4. 10.1038/s41592-019-0430-y 31133757

[56] Sanchez-Vega F, Mina M, Armenia J, et al. Oncogenic signaling pathways in the cancer genome atlas. Cell 2018;173:e10:321–37. 10.1016/j.cell.2018.03.035 PMC6070353 29625050

[57] Böger C, Krüger S, Behrens HM, et al. Epstein-Barr virus-associated gastric cancer reveals intratumoral heterogeneity of PIK3CA mutations. Ann Oncol 2017;28:1005–14. 10.1093/annonc/mdx047 28453696 PMC5406766

[58] Bartok O, Pataskar A, Nagel R, et al. Anti-tumour immunity induces aberrant peptide presentation in melanoma. Nature 2021;590:332–7. 10.1038/s41586-020-03054-1 33328638

[59] Curtis M, Kenny HA, Ashcroft B, et al. Fibroblasts mobilize tumor cell glycogen to promote proliferation and metastasis. Cell Metab 2019;29:141–55. 10.1016/j.cmet.2018.08.007 30174305 PMC6326875

[60] Zhang B, Wu Q, Li B, et al. m^6^A regulator-mediated methylation modification patterns and tumor microenvironment infiltration characterization in gastric cancer. Mol Cancer 2020;19:53. 10.1186/s12943-020-01170-0 32164750 PMC7066851

[61] Subudhi SK, Vence L, Zhao H, et al. Neoantigen responses, immune correlates, and favorable outcomes after ipilimumab treatment of patients with prostate cancer. Sci Transl Med 2020;12:eaaz3577. 10.1126/scitranslmed.aaz3577 32238575

[62] Wang X, Nagl NG, Flowers S, et al. Expression of p270 (ARID1A), a component of human SWI/SNF complexes, in human tumors. Int J Cancer 2004;112:636–42. 10.1002/ijc.20450 15382044

[63] Labadie BW, Bao R, Luke JJ. Reimagining IDO pathway inhibition in cancer immunotherapy via downstream focus on the Tryptophan-Kynurenine-Aryl hydrocarbon axis. Clin Cancer Res 2019;25:1462–71. 10.1158/1078-0432.CCR-18-2882 30377198 PMC6397695

[64] Li H, Bullock K, Gurjao C, et al. Metabolomic adaptations and correlates of survival to immune checkpoint blockade. Nat Commun 2019;10:4346. 10.1038/s41467-019-12361-9 31554815 PMC6761178

[65] Topper MJ, Vaz M, Marrone KA, et al. The emerging role of epigenetic therapeutics in immuno-oncology. Nat Rev Clin Oncol 2020;17:75–90. 10.1038/s41571-019-0266-5 31548600 PMC7254932

[66] Xiao Q, Zhou D, Rucki AA, et al. Cancer-Associated fibroblasts in pancreatic cancer are reprogrammed by tumor-induced alterations in genomic DNA methylation. Cancer Res 2016;76:5395–404. 10.1158/0008-5472.CAN-15-3264 27496707 PMC5026619

[67] Arensman MD, Yang XS, Zhong W, et al. Anti-Tumor immunity influences cancer cell reliance upon ATG7. Oncoimmunology 2020;9:1800162. 10.1080/2162402X.2020.1800162 32923161 PMC7458662

