# Supplementary material for: Tumor microenvironment evaluation promotes precise checkpoint immunotherapy of advanced gastric cancer
Source: J Immunother Cancer. 2021 Aug 10;9(8):e002467. doi: 10.1136/jitc-2021-002467 (PMC8356190; doi:10.1136/jitc-2021-002467)
Supplement: Supplementary data [file jitc-2021-002467supp003.pdf]

Tumor microenvironment evaluation promotes precise checkpoint immunotherapy of advanced gastric cancer

Supplementary Methods

1. Signature Genes reduction

To optimize tumor microenvironment evaluation for more convenient translational medicine, dimension reduction was conducted to choose most predictive genes from 244 TMEscore relevant signature genes which established in our previous study[1]. All signature genes were ordered by the feature importance contributed to prediction accuracy for immune checkpoint blockades response in these datasets[1-3]. Gene importance was calculated as following formula:

$$\text{Feature importance} = \sum \frac{-log10(Predictive\ \ P\ value)}{Involed\ Patients}$$

Genes with feature importance lesser than -90 and larger than +80 (**Fig. S1A**) were selected as signature genes which applied to tumor microenvironment evaluation using PCA methodology[1].

2. Gene expression data resources and preprocessing

Level 3 RNA-Seq data (raw counts) of TCGA Pan-Caner tumor samples were downloaded from the UCSC Xena browser (http://xena.ucsc.edu/) Genomic Data Commons hub. For TCGA patients, corresponding survival data were obtained from supplementary data of published research[4]. Updated clinical and molecular information for TCGA samples were obtained from Genomic Data Commons (https://portal.gdc.cancer.gov/) using the R package TCGAbiolinks[5]. RNA-seq count data were transformed into TPM[6] to conduct the following statistical and differentially expressed gene (DEG) analyses. Raw data from the microarray datasets generated using Affymetrix® and Illumina® were downloaded from the Gene Expression Omnibus (https://www.ncbi.nlm.nih.gov/geo/). The raw data for the dataset from Affymetrix® were processed using the RMA algorithm for background adjustment using the Affy package. RMA was used to perform background adjustment, quantile normalization, and final summarization of oligonucleotides per transcript using the median polish algorithm. The raw data for the dataset from Illumina® were processed using the Lumi package. The ComBat algorithm[7] was applied to reduce the likelihood of batch effects from non-biological technical biases.

3. Tumor microenvironment deconvolution

Computational algorithms and tools used to deconvolute the microenvironment detailed as follows:

ESTIMATE[8]: ImmuneScore, Stromalscore, and tumor purity were assessed computationally in all longitudinal samples using estimates derived from the RNA-seq data using the ESTIMATE algorithm[8] that uses gene expression signatures to infer the fraction of stromal and immune cells in tumor samples.

CIBERSORT[9]: To quantify the proportions of immune cells in the tumor samples, we used the CIBERSORT algorithm and the LM22 gene signature, which allows for highly sensitive and specific discrimination of 22 human immune cell phenotypes including B cells, T cells, NK cells, macrophages, dendritic cells (DCs), and myeloid subsets. CIBERSORT is a deconvolution algorithm that uses a set of reference gene expression values (a signature with 547 genes) considered a minimal representation for each

cell type and, based on those values, infers cell type proportions in data from bulk tumor samples with mixed cell types using support vector regression. Gene expression profiles were prepared using standard annotation files and data were uploaded to the CIBERSORT web portal (http://cibersort.stanford.edu/), with the algorithm run using the LM22 signature at 1,000 permutations.

4. Functional and pathway enrichment analysis

Gene annotation enrichment analysis was performed with the R package clusterProfiler[10]. Enrichment *P*-values were based on 1,000 permutations and subsequently adjusted for multiple testing using the Benjamini-Hochberg procedure to control the false discovery rate (FDR)[11]. Gene Ontology (GO) and KEGG terms were identified with a strict cutoff of $P < 0.01$ and an FDR of less than 0.05. We also identified pathways that were up- and down-regulated among groups by running a gene set enrichment analysis (GSEA)[12] of the adjusted expression data for all transcripts.

5. Other data resources

① Genomic, transcriptomic, and matched clinical data from patients with metastatic urothelial cancer treated with anti-PD-L1 agent (atezolizumab)[13] is available under the Creative Commons 3.0 license and can be downloaded from http://research-pub.gene.com/IMvigor210CoreBiologies.

② The numbers of predicted neo-antigens based on tumor-specific HLA typing, total mutations and CYT for each patient were obtained for 263 STAD samples from Supplementary Table S4 from Rooney *et al.*[14].

③ The abundance of viruses detected in TCGA-PanCaner cohorts was obtained from research of Cao *et.al* [15].

6. Other gene signatures enrolled in this study

To characterize the metabolism, immune microenvironment and other prevalent gene signatures activation in each tumor samples, PCA algorithm was apply to determine the pathway activity using gene sets (summarized in IOBR package: https://github.com/IOBR/IOBR) curated by Mariathasan *et al.*[2], Cristescu *et al.*[16], Rooney *et al.*[14], and Rosario *et al.*[17]. Gene sets curated by Mariathasan *et al.*[2], contain : A: CD8 T-effector signature[18]; B: Antigen processing machinery[19]; C: Immune checkpoint; D: Epithelial-mesenchymal transition (EMT) markers previously reported[20]; E: Pan-fibroblast TGF-β response signature (Pan-F-TBRS) [2]; F: DNA replication-dependent histones[2]; G: Select members of the DDR-relevant gene set [21]; H: Angiogenesis signature previously reported [22]; I: Cell cycle genes (KEGG); J: WNT targets[23]; K: Cell cycle regulators[24]; L: Mismatch repair (KEGG); M: Nucleotide excision repair (KEGG); N: Homologous recombination (KEGG). We thereby obtained, for each signature, an enrichment score per sample that indicated the extent of upregulation or downregulation of the associated genes. A minimum overlap of two genes was required.

References

1. Zeng D, Li M, Zhou R, et al. Tumor Microenvironment Characterization in Gastric Cancer Identifies Prognostic and Immunotherapeutically Relevant Gene Signatures. Cancer Immunology Research 2019;**7**(5):737-50 doi: 10.1158/2326-6066.CIR-18-0436[published Online First: Epub Date]|.

2. Mariathasan S, Turley SJ, Nickles D, et al. TGFbeta attenuates tumour response to PD-L1 blockade by contributing to exclusion of T cells. Nature 2018;**554**(7693):544-48 doi: 10.1038/nature25501[published Online First: Epub Date]|.
3. Hugo W, Zaretsky JM, Sun L, et al. Genomic and Transcriptomic Features of Response to Anti-PD-1 Therapy in Metastatic Melanoma. Cell 2016;**165**(1):35-44 doi: 10.1016/j.cell.2016.02.065[published Online First: Epub Date]|.
4. Liu J, Lichtenberg T, Hoadley KA, et al. An Integrated TCGA Pan-Cancer Clinical Data Resource to Drive High-Quality Survival Outcome Analytics. Cell 2018;**173**(2):400-16 e11 doi: 10.1016/j.cell.2018.02.052[published Online First: Epub Date]|.
5. Colaprico A, Silva TC, Olsen C, et al. TCGAbiolinks: an R/Bioconductor package for integrative analysis of TCGA data. Nucleic Acids Research 2016;**44**(8):e71-e71 doi: 10.1093/nar/gkv1507[published Online First: Epub Date]|.
6. Wagner GP, Kin K, Lynch VJ. Measurement of mRNA abundance using RNA-seq data: RPKM measure is inconsistent among samples. Theory in Biosciences 2012;**131**(4):281-85 doi: 10.1007/s12064-012-0162-3[published Online First: Epub Date]|.
7. Johnson WE, Li C, Rabinovic A. Adjusting batch effects in microarray expression data using empirical Bayes methods. Biostatistics 2007;**8**(1):118-27 doi: 10.1093/biostatistics/kxj037[published Online First: Epub Date]|.
8. Yoshihara K, Shahmoradgoli M, Martinez E, et al. Inferring tumour purity and stromal and immune cell admixture from expression data. Nat Commun 2013;**4**:2612 doi: 10.1038/ncomms3612[published Online First: Epub Date]|.
9. Newman AM, Liu CL, Green MR, et al. Robust enumeration of cell subsets from tissue expression profiles. Nat Methods 2015;**12**(5):453-7 doi: 10.1038/nmeth.3337[published Online First: Epub Date]|.
10. Yu G, Wang LG, Han Y, He QY. clusterProfiler: an R package for comparing biological themes among gene clusters. OMICS 2012;**16**(5):284-7 doi: 10.1089/omi.2011.0118[published Online First: Epub Date]|.
11. Benjamini Y, Hochberg Y. Controlling the False Discovery Rate: A Practical and Powerful Approach to Multiple Testing. Journal of the Royal Statistical Society: Series B (Methodological) 1995;**57**(1):289-300 doi: 10.1111/j.2517-6161.1995.tb02031.x[published Online First: Epub Date]|.
12. Subramanian A, Tamayo P, Mootha VK, et al. Gene set enrichment analysis: A knowledge-based approach for interpreting genome-wide expression profiles. Proceedings of the National Academy of Sciences 2005;**102**:15545-50
13. Mariathasan S, Turley SJ, Nickles D, et al. TGFbeta attenuates tumour response to PD-L1 blockade by contributing to exclusion of T cells. Nature 2018;**554**(7693):544-48 doi: 10.1038/nature25501[published Online First: Epub Date]|.
14. Rooney MS, Shukla SA, Wu CJ, Getz G, Hacohen N. Molecular and genetic properties of tumors associated with local immune cytolytic activity. Cell 2015;**160**(1-2):48-61 doi: 10.1016/j.cell.2014.12.033[published Online First: Epub Date]|.
15. Cao S, Wendl MC, Wyczalkowski MA, et al. Divergent viral presentation among human tumors and adjacent normal tissues. Sci Rep 2016;**6**:28294 doi: 10.1038/srep28294[published Online First: Epub Date]|.
16. Cristescu R, Mogg R, Ayers M, et al. Pan-tumor genomic biomarkers for PD-1 checkpoint blockade-based immunotherapy. Science 2018;**362**(6411) doi: 10.1126/science.aar3593[published Online First: Epub Date]|.
17. Rosario SR, Long MD, Affronti HC, Rowsam AM, Eng KH, Smiraglia DJ. Pan-cancer analysis of transcriptional metabolic dysregulation using The Cancer Genome Atlas. Nat Commun 2018;**9**(1):5330 doi: 10.1038/s41467-018-07232-8[published Online First: Epub Date]|.
18. Rosenberg JE, Hoffman-Censits J, Powles T, et al. Atezolizumab in patients with locally advanced and metastatic urothelial carcinoma who have progressed following treatment with platinum-based chemotherapy: a single arm, phase 2 trial. Lancet (London, England) 2016;**387**(10031):1909-20 doi: 10.1016/S0140-6736(16)00561-4[published Online First: Epub Date]|.
19. Şenbabaoğlu Y, Gejman RS, Winer AG, et al. Tumor immune microenvironment characterization in clear cell renal cell carcinoma identifies prognostic and immunotherapeutically relevant messenger RNA signatures. Genome Biology 2016;**17**(1) doi: 10.1186/s13059-016-1092-z[published Online First: Epub Date]|.
20. Damrauer JS, Hoadley Ka Fau - Chism DD, Chism Dd Fau - Fan C, et al. Intrinsic subtypes of high-grade bladder cancer reflect the hallmarks of breast cancer biology. Proc Natl Acad Sci 2014(1091-6490 (Electronic))
21. Lange SS, Takata K-i, Wood RD. DNA polymerases and cancer. Nature Reviews Cancer 2011;**11**:96 doi: 10.1038/nrc2998[published Online First: Epub Date]|.

22. Sjodahl G, Lauss M Fau - Lovgren K, Lovgren K Fau - Chebil G, et al. A molecular taxonomy for urothelial carcinoma. Clin Cancer Res 2012(1078-0432 (Print))
23. Spranger S, Bao R, Gajewski TF. Melanoma-intrinsic beta-catenin signalling prevents anti-tumour immunity. Nature 2015(1476-4687 (Electronic))
24. Cancer Genome Atlas Research N. Comprehensive molecular characterization of urothelial bladder carcinoma. Nature 2014;**507**(7492):315-22 doi: 10.1038/nature12965[published Online First: Epub Date]|.
