# Supplementary material for: Tumor microenvironment evaluation promotes precise checkpoint immunotherapy of advanced gastric cancer
Source: J Immunother Cancer. 2021 Aug 10;9(8):e002467. doi: 10.1136/jitc-2021-002467 (PMC8356190; doi:10.1136/jitc-2021-002467)
Supplement: Supplementary data [file jitc-2021-002467supp002.pdf]

Supplementary Figures for

Tumor microenvironment evaluation promotes precise checkpoint immunotherapy of advanced gastric cancer

The PDF file includes:

Fig. S1. TMEscore predicts ICB therapeutic response and prognosis.
Fig. S2. ROC analyses of TMEscore highlight its predictive value for the anti-PD-1 response, MSI-H, and EBV status.
Fig. S3. Analyses in TCGA-STAD and ACRG cohorts corroborate the predictive value of the TMEscore.
Fig. S4. TMEscore correlates with tumor-related viral infections and immune activation.
Fig. S5. Significantly higher TMB or neoantigen load are not observed in viral infected tumors in Pan-Cancer analyses
Fig. S6. *ARID1A* and *PIK3CA* deficiency is significantly related to the high TMEscore.
Fig. S7. Mutations of *ARID1A* and *PIK3CA* are enriched in the in EBV and MSI subtype
Fig. S8. *ARID1A* deficiency is associated with activated anti-tumor immunity.
Fig. S9. Pathway mutations primarily accumulate in the high-TME fraction
Fig. S10. ACRG cohort verifies the correlations among kynurenine metabolism, glycogen metabolism, and TMEscore
Fig. S11. Methylation of *VAMP8 and ATG7* exhibit diverse correlations with immune activity.

Fig. S1 TMEscore predicts ICB therapeutic response and prognosis.

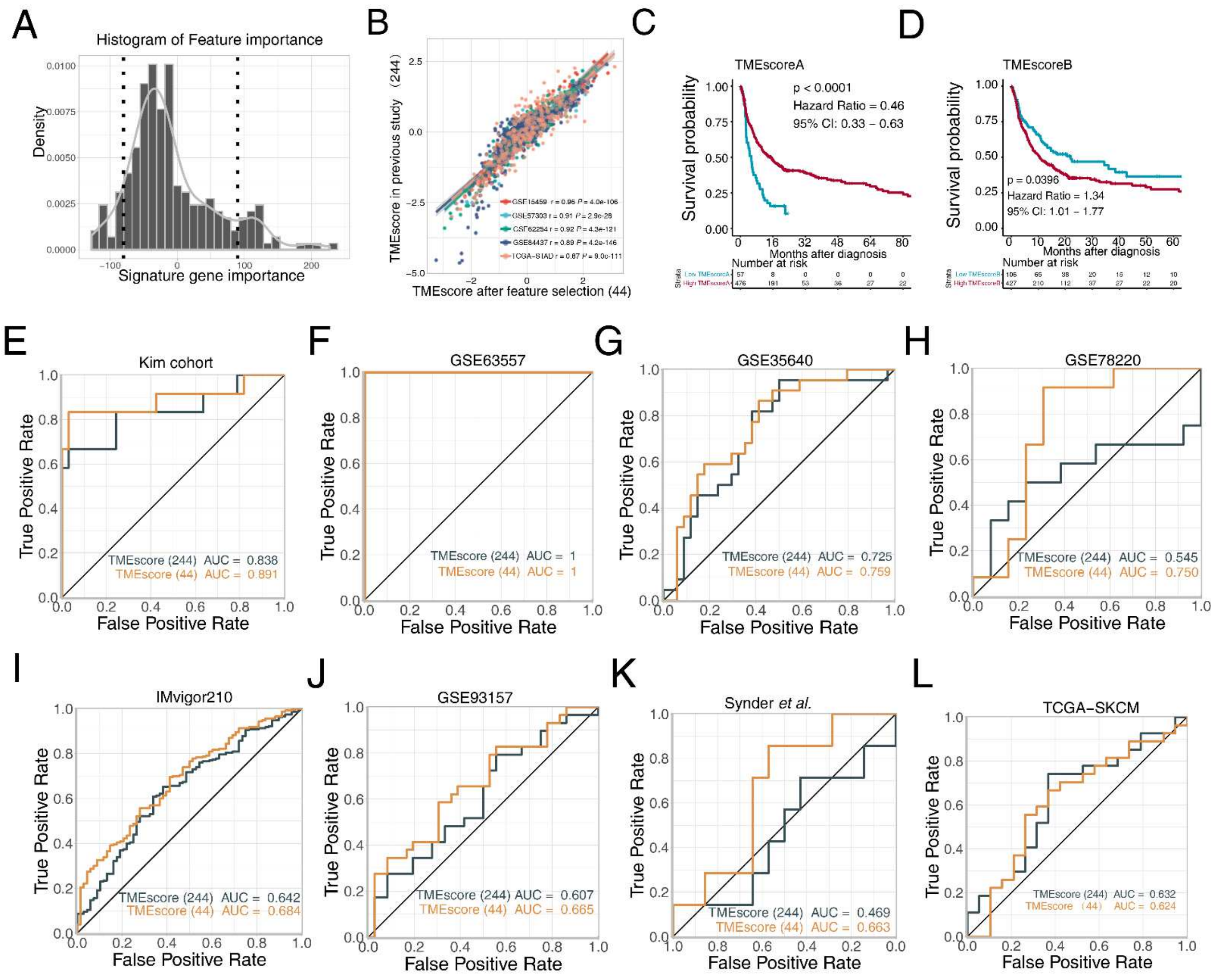

A. Histogram of feature importance displayed the results of feature engineering conducted to reduce TMEscore signature genes. The cutoff was set at -90 and 90.

B. The dotplot exhibited a strong correlation between the TMEscore calculated before and after dimension reduction. The x and y axis respectively represent the TMEscore generated with 254 genes (before dimension reduction) and 44 genes (after). Every single dot represents one patient, and distinctive cohorts are identified in different colors. GSE15459 (red): r = 0.96, *P* = 4.0e-106; GSE57303 (blue): r = 0.91, *P* = 2.9e-28; GSE62254 (green): r = 0.92, *P* = 4.3e-121; GSE84437 (purple): r = 0.89, *P* = 4.2e-146; TCGA-STAD (orange): r = 0.87, *P* = 9.0e-111.

C-D. Kaplan-Meier survival analysis supported the prognosis predictive value of TMEscoreA (**G**) and TMEscoreB (**H**). High (red) TMEscoreA (*P* < 0.0001, Hazard Ratio = 0.46, 95% CI: 0.33 − 0.63) and low (blue) TMEscoreB (*P* = 0.0396, Hazard Ratio = 1.34, 95% CI: 1.01 – 1.77) was associated with more favorable overall survival in study of multiple meta-data.

E-L. Comparison of predictive capacity among TMEscore before and after gene reduction in eight independent IO-cohorts. ROC analyses suggested the TMEscore (44 genes) harboring a higher AUC when compared with TMEscore (244 genes) in the Kim cohort (**E**) (AUC = 0.891 and 0.838), the GSE63557 (**F**) (AUC = 1 and 1), the GSE35640 (**G**) (AUC = 0.759 and 0.725), the GSE78220 (**H**) (AUC = 0.750 and 0.545), the IMvigor210 cohorts (**I**) (AUC = 0.684 and 0.642), the GSE93157 (**J**) (AUC = 0.665 and 0.607), the Synder *et al.* (**K**) (AUC = 0.663 and 0.469) and the TCGA-SKCM cohorts (**L**) (AUC = 0.624 and 0.632).

Fig. S2 ROC analyses of TMEscore highlight its predictive value for anti-PD-1 response, MSI-H and EBV status.

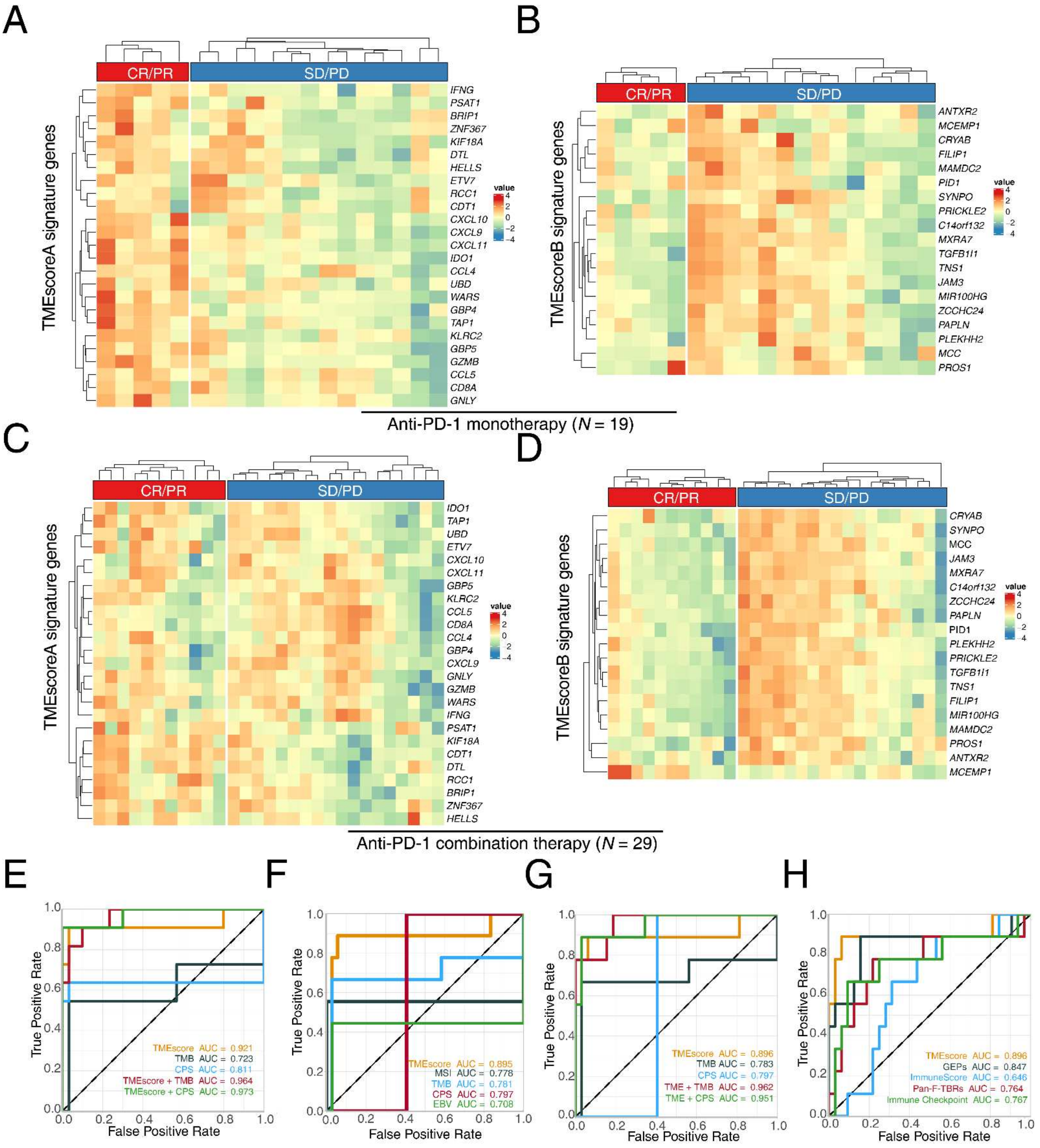

A-D. Heatmaps displayed the signature genes expression of TMEscoreA (**A**) and TMEscoreB (**B**) respectively, in the responsive (CR/PR) and the progressive (SD/PD) gastric cancer in the context of anti-PD-1 monotherapy. Consistent results of TMEscoreA (**C**) and TMEscoreB pertinent genes (**D**) in the anti-PD-1 combination therapy setting were also exhibited. CR: Complete Response; PR: Partial Response; SD: Stable Disease; PD: Progressive Disease.

E. ROC analyses indicated that combination with TMB and CPS slightly elevated the AUC of TMEscore alone (TMEscore, TMB, CPS, TMEscore + TMB, TMEscore + CPS, AUC = 0.921, 0.723, 0.811, 0.964, 0.973, respectively), but the statistical significance was not observed, using pairwise comparison. Detail *P* values are available in Table S7.

F-H. TMEscore harbored highest AUC in predicting MSI and EBV molecular subtype, in comparison with MSI status, TMB, CPS, and EBV status (AUC = 0.895, 0.778, 0.781, 0.797, 0.708, respectively;) (**F**). Its collaboration with TMB or CPS,

slightly but not statistically increased the AUC (TMEscore, TMEscore + TMB, TMEscore + CPS, AUC = 0.896, 0.962, 0.951, respectively) (**G**). Moreover, predictive AUC of TMEscore also superior than other prevalent transcriptome-based signatures, comprising gene expression profile score (GEPs), Immunoscore, pan-fibroblast TGFβ response signature (Pan-F-TBRs), and Immune checkpoint (AUC = 0.896, 0.847, 0.646, 0.764, 0.767, respectively) (**H**). Corresponding *P* values of pair comparison test please see Table S7.

Fig. S3 Analyses in TCGA-STAD and ACRG cohort corroborate the predictive value of TMEscore.

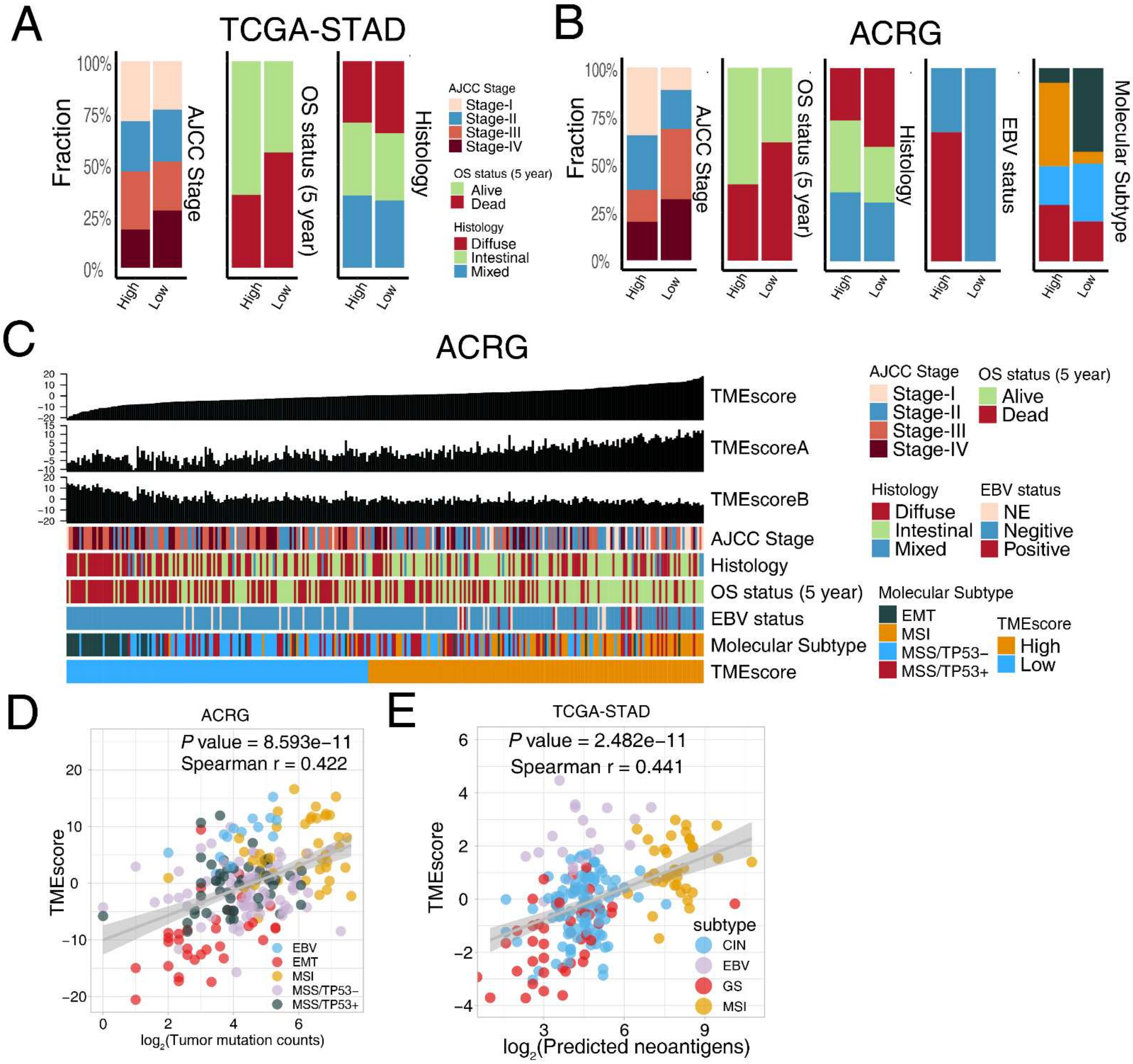

A-B. Barplots displayed the clinicopathological features distributions in high- and low-TMEscore fraction in TCGA-STAD (**A**) and ACRG cohort (**B**). EBV positive and MSI-H status are predominantly enriched in high TMEscore subgroup.

C. For each patient (columns) in ACRG cohort, landscape of clinicalpathological features and molecular characterizations were exhibited. Column annotations represent AJCC stage (stage I, II, III, IV); five-year survival (alive, dead); histology (diffuse, intestinal, mixed); EBV status (negative, positive, NE); molecular subtype (EMT, MSI, MSS/TP53-, MSS/TP53+); and TME subtype (high, low), for each sample. TMEscore, TMEscoreA and TMEscoreB are displayed at the top of the panel. Results of ACRG cohort confirmed that EBV positive and MSI-H patients are enriched in high TMEscore fraction.

D-E. Dotplots supported close correlation between TMB and TMEscore in ACRG (**D**) and btween neoantigen and TMEscore in TCGA-STAD (**E**) cohort. Every single dot represents one sample, corresponding molecular subtypes were identified in different colors in ACRG cohort (EBV: pink, EMT: red, MSI: blue, MSS/TP53-: yellow, MSS/TP53+: green; Spearman test,

r = 0.441, *P* = 2.5e-11) and TCGA-STAD cohort (CIN: blue, EBV: pink, GS: red, MSI: yellow; Spearman test, r = 0.422, *P* = 8.6e-11).

Fig. S4 TMEscore correlates with tumor-related viral infections and immune activation.

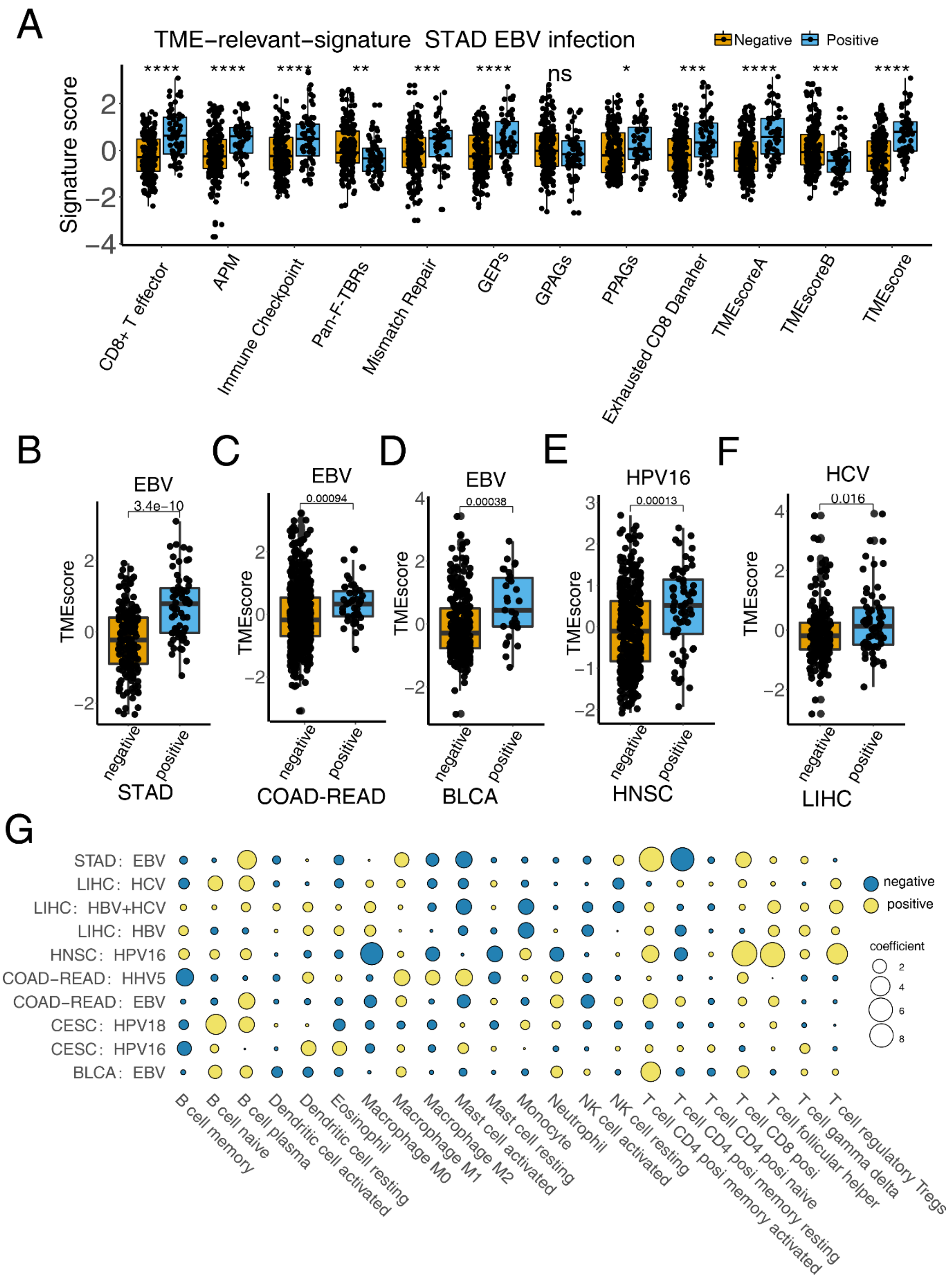

A. Boxplot demonstrated an increase of TMEscore and TME relevant signatures including CD8+T effector, APM, Immune checkpoint, mismatch repair, GEPs, and PPAGs, whereas pan-F-TBRs and GPAGs decreased in EBV positive groups (blue), compared with the EBV negative setting (yellow). *P* value were shown with ****, ***, **, *, ns, represent $P < 0.0001$, $P < 0.001$, $P < 0.01$, $P < 0.05$, $P > 0.05$, respectively.

B-F. Boxplots indicated that TMEscore was significantly elevated in cancers with viral infections, comprising EBV in TCGA-STAD (*P* = 3.4e-10), The Cancer Genome Atlas Colon adenocarcinoma Rectum adenocarcinoma Esophageal carcinoma

(TCGA-COAD-READ) (*P* = 1.6e-02) and The Cancer Genome Atlas Bladder Urothelial Carcinoma (TCGA-BLCA) (*P* = 9.4e-04); HCV in The Cancer Genome Atlas Liver hepatocellular carcinoma (TCGA-LIHC) (*P* = 3.8e-04); HPV16 in The Cancer Genome Atlas Head and Neck squamous cell carcinoma (TCGA-HNSC) (*P* = 1.3-04). *P* values were calculated using Wilcoxon test.

G. Corplot exhibited the coefficients among aforementioned tumor-associated viral infections and a variety of TME immune cell signatures. The coefficient was represented using the size of the dot, while colors yellow (positive) and blue (negative) marked its orientation.

Fig. S5 Significantly higher TMB are not observed in viral infected tumors in Pan-Cancer analyses

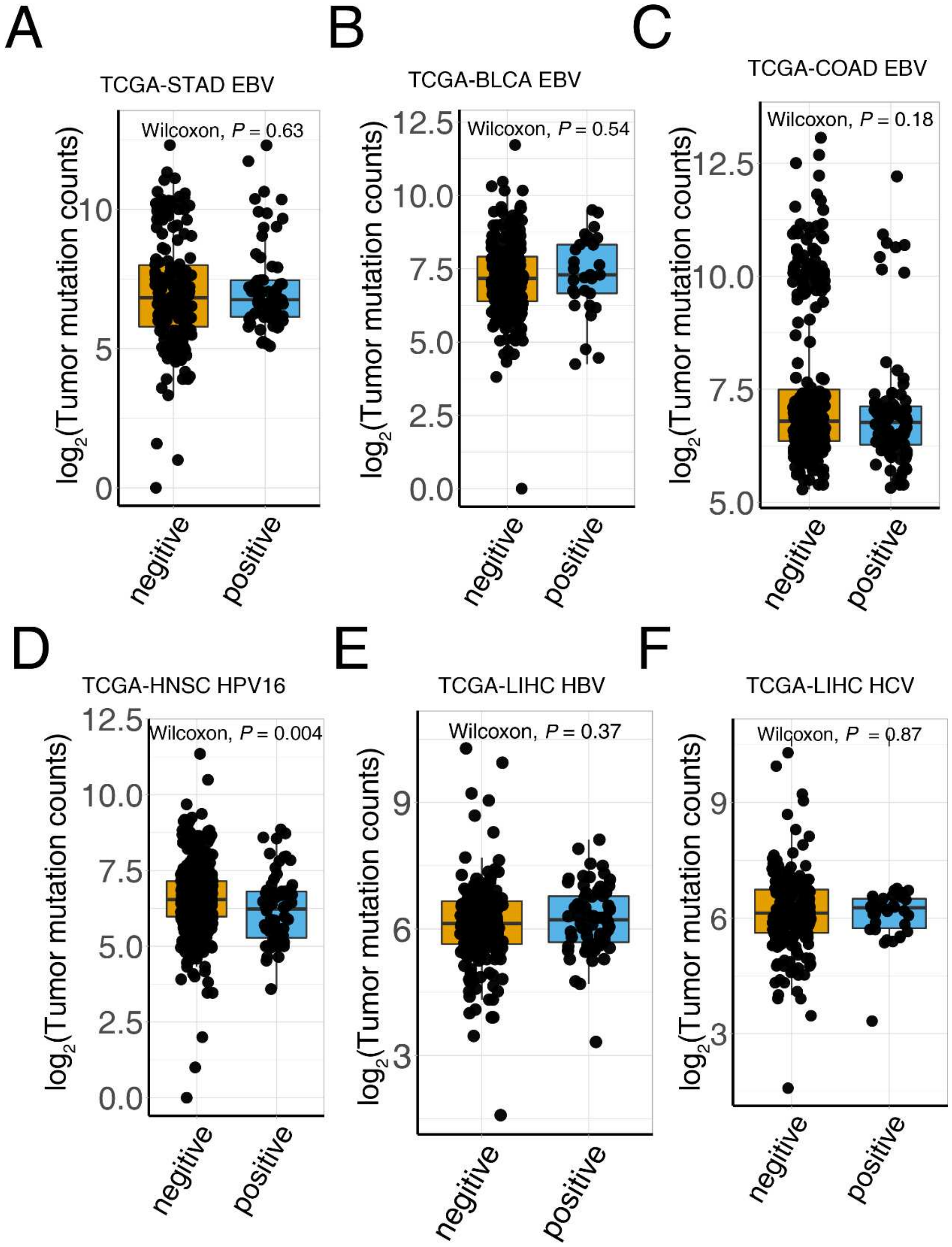

A-F. Boxplots show bare statistical elevation of tumor mutation load in viral infected tumors, including EBV in TCGA-STAD (*P* = 0.63) (**A**), TCGA-BLCA (*P* = 0.54) (**B**), TCGA-COAD-READ (*P* = 0.18) (**C**); HPV16 in TCGA-HNSC (*P* = 0.004) (**D**), HBV (*P* = 0.37) (**E**) and HCV in (TCGA-LIHC) (*P* = 0.87) (**F**). *P* values were calculated using Wilcoxon test.

Fig. S6 *ARID1A* and *PIK3CA* deficiency is significantly related to high TMEscore.

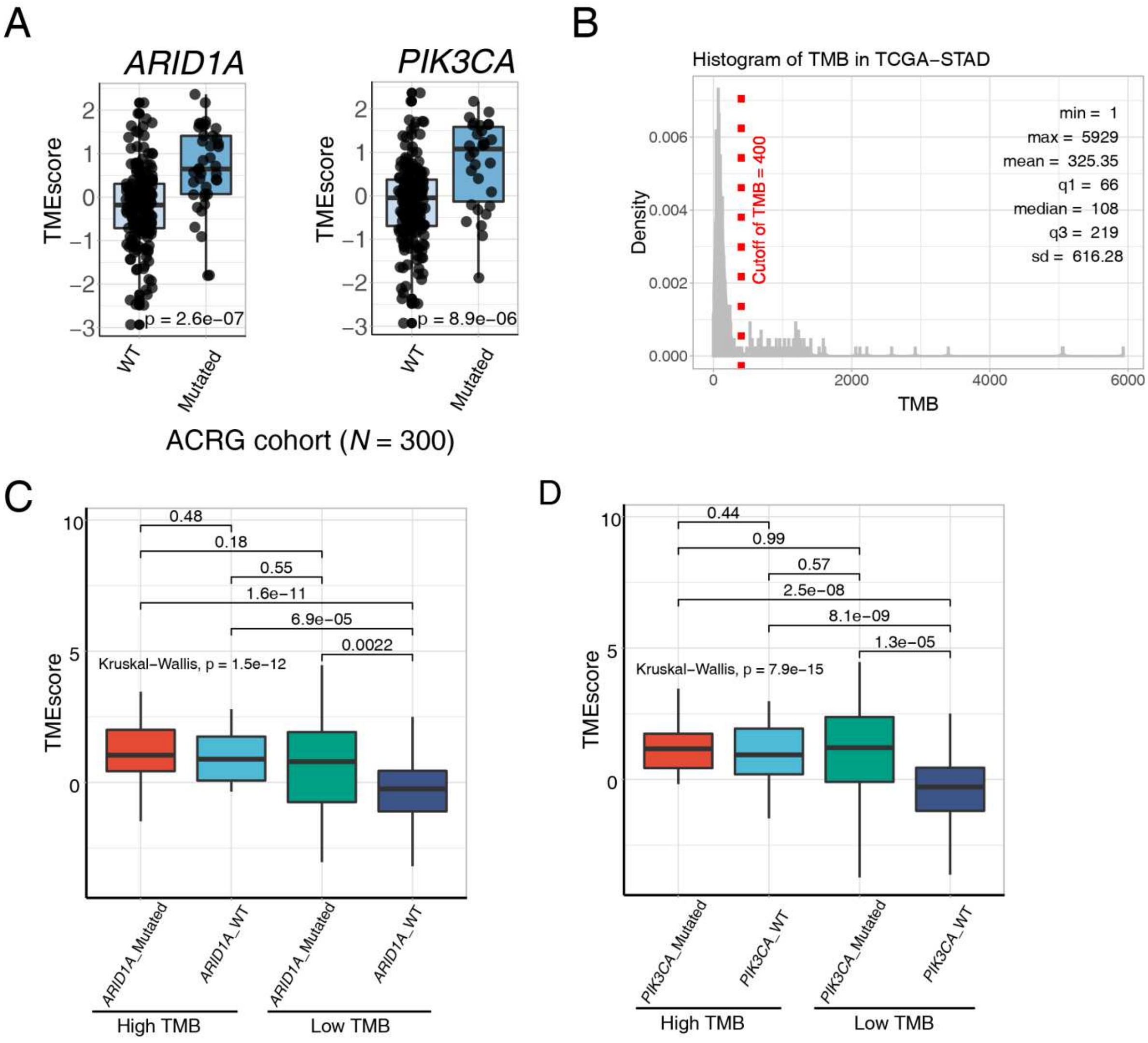

A. Analysis of ACRG cohort corroborated that *ARID1A* (Wilcoxon, *P* = 2.6e-07) and *PIK3CA* (Wilcoxon, *P* = 8.9e-06) deficiencies harbored higher TMEscore.

B. Histogram of TMB in TCGA-STAD cohort. The cutoff of TMB was set at 400 according to the distribution of TMB and previous research.

C. Diverse distribution of TMEscore of *ARID1A* mutations in high TMB and low TMB settings was displayed (Kruskal Wallis test, *P* = 1.5e-12). TMEscore significantly increased in *ARID1A* mutations in low TMB setting (Wilcoxon, *P* = 0.0022).

D. Diverse distribution of TMEscore of *PIK3CA* mutations in high TMB and low TMB settings was displayed (Kruskal Wallis test, *P* = 7.9e-15). TMEscore significantly increased in *PIK3CA* mutations in low TMB setting (Wilcoxon, *P* = 1.3e-05).

Fig. S7 Mutations of *ARID1A* and *PIK3CA* are enriched in the in EBV and MSI subtype

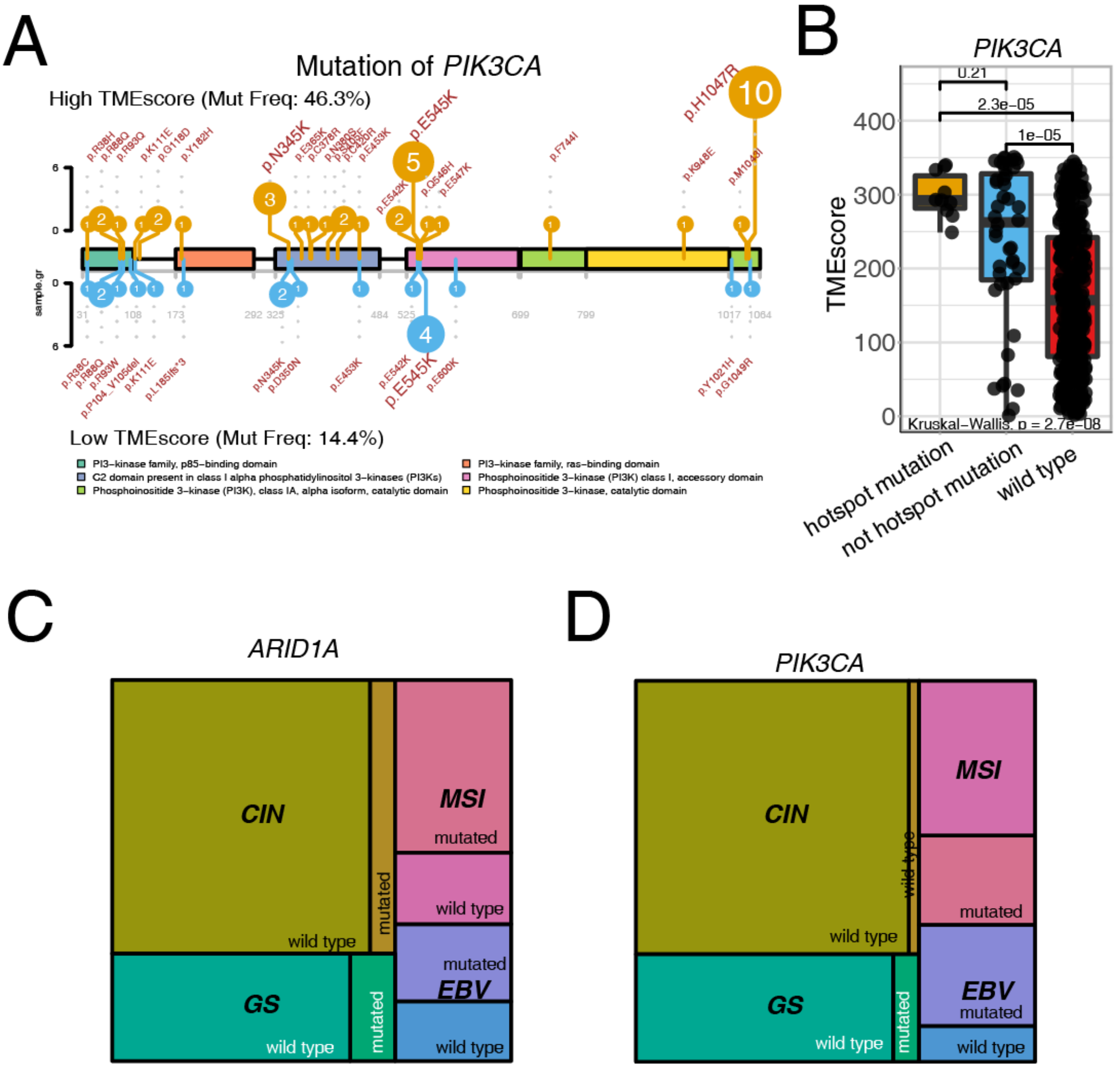

A-B. The landscape of the *PIK3CA* deficient mutation positions and corresponding TMEscore was displayed. p.H1047R mutations were prominently enriched in high-TMEscore subset. The mutation rates of high- (yellow) and low- (blue) TMEscore are shown (**A**). *PIK3CA* deficiency is correlated with the higher TMEscore despite no statistical discrepancy was observed between hotspot and non-hotspot mutation of *PIK3CA* (Kruskal Wallis test, P = 9e-11) (**B**).

C-D. Mutation rate of *ARID1A* (**C**) and *PIK3CA* (**D**) are elevated in EBV and MSI molecular subtypes than that in CIN and GS subtype in TCGA-STAD cohort. The P value was calculated using Chi-Squire test (*ARID1A*: P < 2.2e-16; *PIK3CA*: P < 2.2e-16).

Fig. S8 ***ARID1A*** **deficiency is associated with activated anti-tumor immunity.**

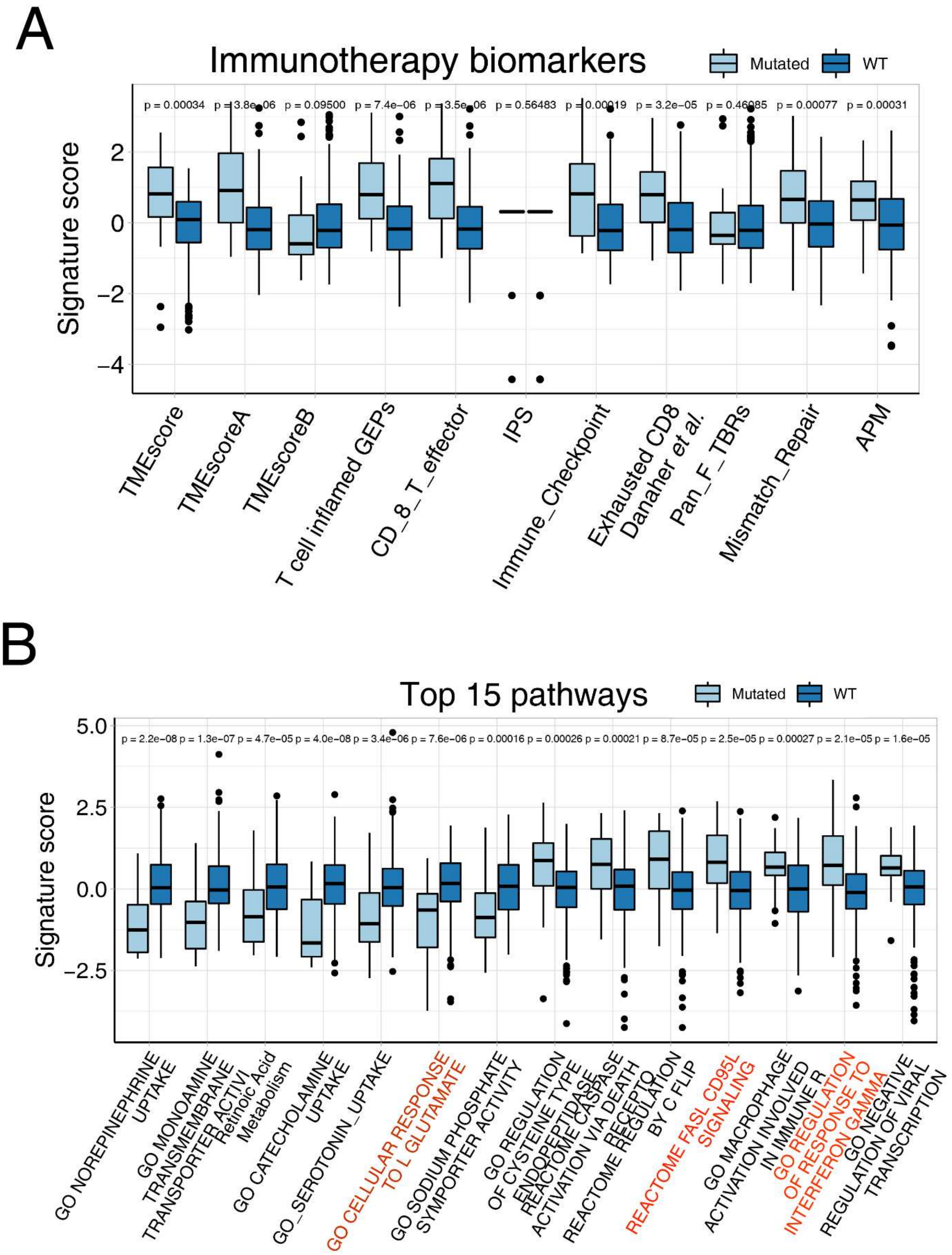

A. TME-related and immune-activated signature score was significantly increased in *ARID1A* deficient setting (light blue), in comparison with that in the wild type (navy blue). *P* values were shown.

B. Metabolic signature scores were significantly decreased in *ARID1A* deficient setting (light blue) compared with that in the wild type (navy blue), while tumor and immune-related signature scores increased in *ARID1A* deficient setting. *P* values were shown.

Fig. S9 Pathway mutations primarily accumulates in high-TME fraction

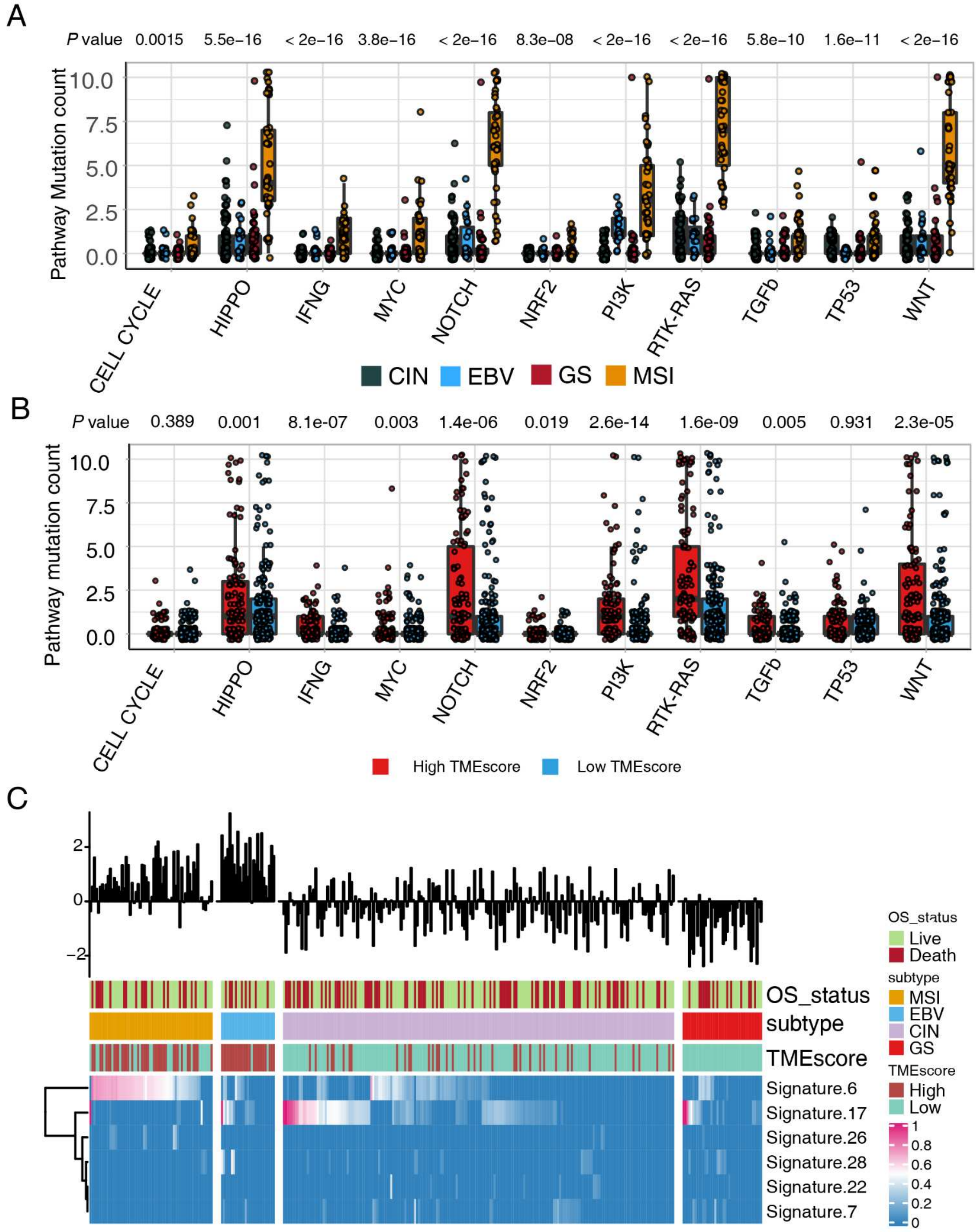

A. High mutation counts were observed both in EBV and MSI molecular subtypes when comparison was performed in PI3K pathway.

B. The discrepancy of mutation frequency of each pathway in high (red) and low (blue) TMEscore subsets was displayed. PI3K pathway mutation counts were markedly elevated in patients with high-TMEscore (Wilcoxon test, $P = 2.6e\text{-}14$).

C. For each sample (columns), profile of mutation signatures in the Catalogue Of Somatic Mutations In Cancer (COSMIC) (rows) were characterized. Column annotations represent OS status (live, dead), molecular subtype (CIN, EBV, GS, MSI); and TMEscore (high, low). TMEscore was displayed in the top panel. mismatch repair associated signature 6 was correlated with high TMEscore. Detail information see table S12. Colors (blue to red) represents the corresponding expression levels (low to high).

Fig. S10 ACRG cohort verifies the correlations among kynurenine metabolism, glycogen metabolism, and TMEscore

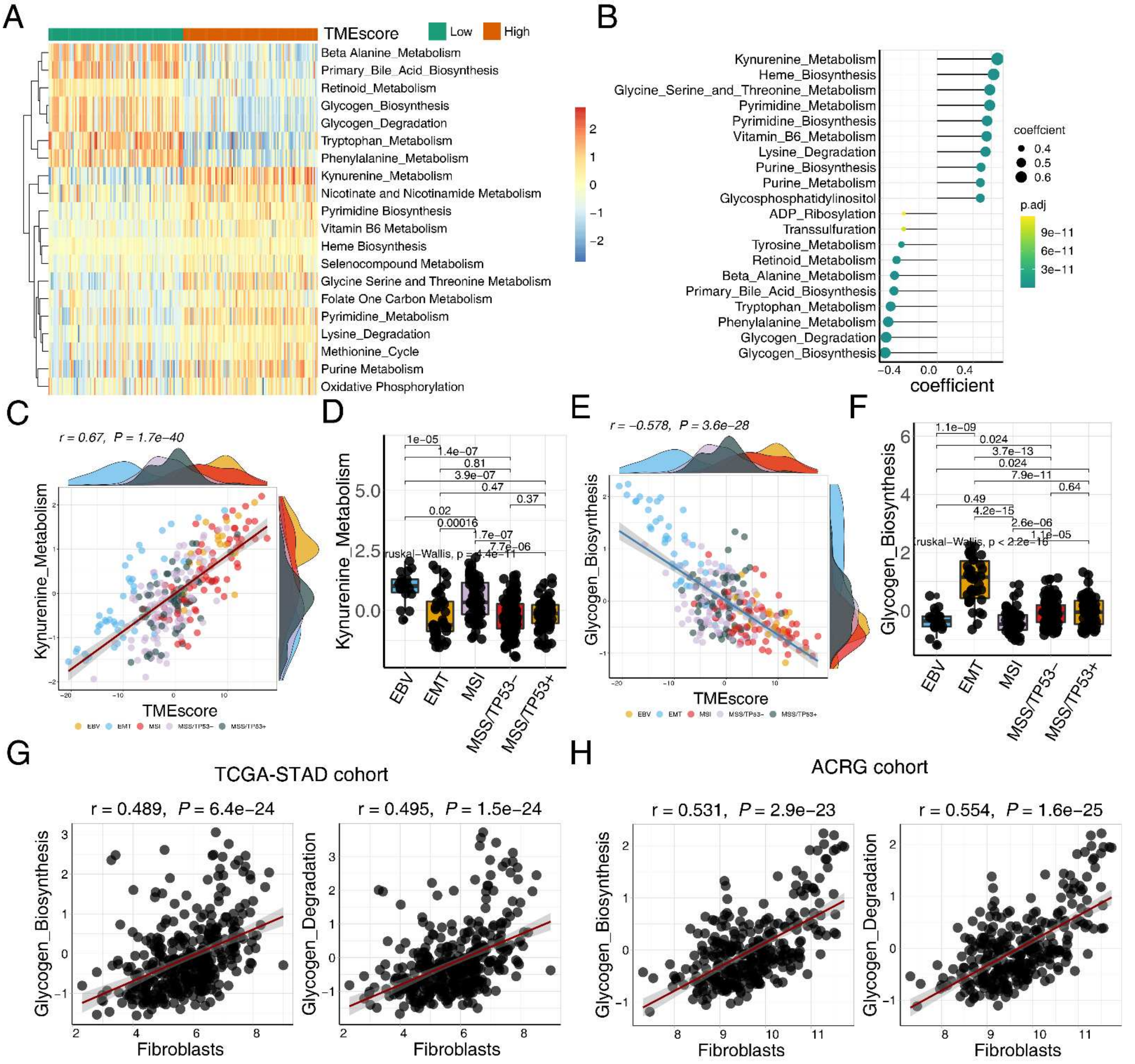

A. The transcriptomic profile of metabolic pathway was exhibited in ACRG cohort. For each patient (columns), signaling pathways (rows) were characterized in the heatmap. the Column annotations high- (orange) and low- (green) TMEscore. Colors (blue to red) represents the corresponding expression levels (low to high).

B. The correlation between TMEscore and metabolism signatures were displayed. The size of dot represents coefficient.

C. A close correlation was observed between TMEscore and kynurenine metabolism. Every single dot represents one sample, corresponding molecular subtypes were identified in different colors (EBV: yellow, EMT: blue, MSI: red, MSS/TP53-: pink, MSS/TP53+: dark; Spearman test, r = 0.67, *P* = 1.7e-40).

D. Kynurenine metabolism was significantly upregulated in EBV and MSI subtype (Kruskal Wallis test, *P* = 4.4e-11).

E. A negative correlation was observed between TMEscore and glycogen biosynthesis. Every single dot represents one sample, corresponding molecular subtypes were identified in different colors (EBV: yellow, EMT: blue, MSI: red, MSS/TP53-: pink, MSS/TP53+: dark; Spearman test, r = -0.578, *P* = 3.6e-28).

F. Glycogen biosynthesis was significantly upregulated in EMT subtype (Kruskal Wallis test, *P* < 2.2e-16).

G. Fibroblasts was significantly positively correlated with glycogen biosynthesis and glycogen degradation in TCGA-STAD cohort (glycogen biosynthesis: Spearman test, r = 0.489, *P* = 6.4e-24; glycogen degradation: Spearman test, r = 0.495, *P* =

1.5e-24).

H. Fibroblasts was significantly positively correlated with glycogen biosynthesis and glycogen degradation in ACRG cohort (glycogen biosynthesis: Spearman test, r = 0.531, *P* = 2.9e-23; glycogen degradation: Spearman test, r = 0.554, P = 1.6e-25).

Fig. S11 Methylation of *VAMP8 and ATG7* exhibit diverse correlations with immune activity.

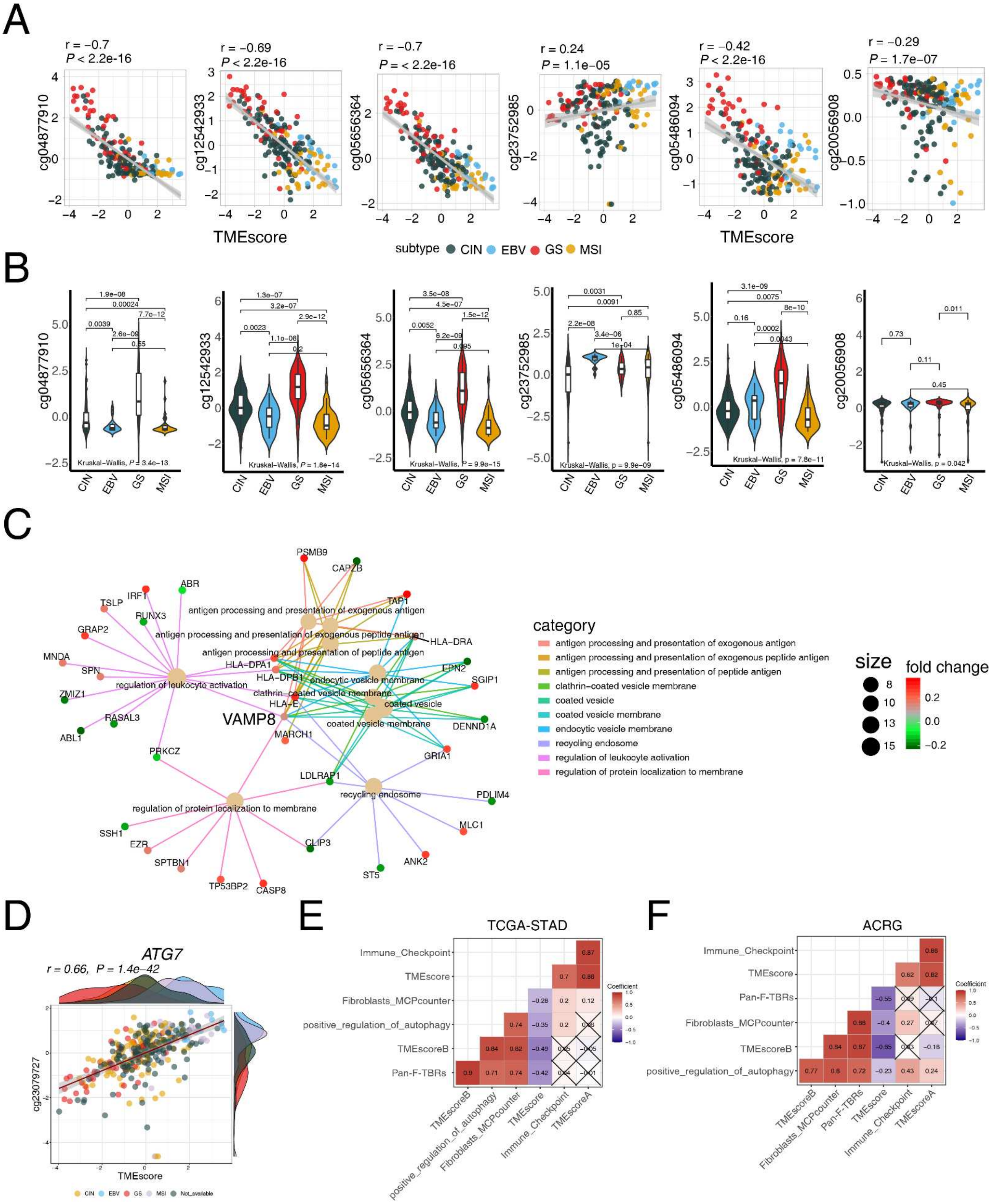

A. cg04877910 (r = -0.7, *P* < 2.2e-16), cg12542933 (r = -0.69, *P* < 2.2e-16), cg05656364 (r = -0.7, *P* < 2.2e-16), cg05486094 (r = -0.42, *P* < 2.2e-16) and cg20056908 (r = -0.29, *P* = 1.7e-07) of *VAMP8* methylation were negatively associated with high TMEscore, whereas cg23752985 (r = 0.24, *P* = 1.1e-05) region exhibited an inverse trend. Every single dot represents one

sample, corresponding molecular subtypes were identified in different colors (CIN: dark, EBV: blue, GS: red, MSI: yellow; Spearman test).

B. cg04877910 (*P* = 3.4e-13), cg12542933 (*P* = 1.8e-14), cg05656364 (*P* = 9.9e-15), cg23752985 (*P* = 9.9e-09), cg05486094 (*P* = 7.8e-11) and cg20056908 (*P* = 0.042) of VAMP8 methylation harbored diverse distribution of molecular subtypes. *P* values were calculated via Kruskal Wallis test.

C. Enrichment of differentially methylated genes was conducted. Regulated pathways associated with *VAMP8* methylations are displayed. Annotation of corresponding pathways are shown, characterized by colors.

D. Cg23079727 of *VAMP8* methylation was positively associated with TMEscore. Every single dot represents one sample, corresponding molecular subtypes were identified in different colors (CIN: yellow, EBV: blue, GS: red, MSI: pink, Not available: dark; Spearman test, r = 0.66, *P* = 1.4e-42).

E. Correlation matrix of *ATG7* related signatures and TMEscore of TCGA-STAD cohort. Coefficients are characterized in number. Colors red and purple represent positive and negative correlations.

F. Correlation matrix of *ATG7* related signatures and TMEscore of ACRG cohort. Coefficients are characterized in number. Colors red and purple represent positive and negative correlations.
